# Heating of hip joint implants in MRI: The combined effect of RF and switched‐gradient fields

**DOI:** 10.1002/mrm.28666

**Published:** 2021-01-22

**Authors:** Alessandro Arduino, Umberto Zanovello, Jeff Hand, Luca Zilberti, Rüdiger Brühl, Mario Chiampi, Oriano Bottauscio

**Affiliations:** ^1^ Istituto Nazionale di Ricerca Metrologica (INRIM) Torino Italy; ^2^ School of Biomedical Engineering and Imaging Sciences King’s College London London United Kingdom; ^3^ Physikalisch‐Technische Bundesanstalt Braunschweig and Berlin Germany

**Keywords:** gradient coil heating, hip prosthesis, MRI safety, numerical simulation, radiofrequency heating

## Abstract

**Purpose:**

To investigate how the simultaneous exposure to gradient and RF fields affects the temperature rise in patients with a metallic hip prosthesis during an MRI session.

**Methods:**

In silico analysis was performed with an anatomically realistic human model with CoCrMo hip implant in 12 imaging positions. The analysis was performed at 1.5 T and 3 T, considering four clinical sequences: turbo spin‐echo, EPI, gradient‐echo, and true fast imaging sequence with steady precession. The exposure to gradient and RF fields was evaluated separately and superposed, by adopting an ad hoc computational algorithm. Temperature increase within the body, rather than specific absorption rate, was used as a safety metric.

**Results:**

With the exception of gradient‐echo, all investigated sequences produced temperature increases higher than 1 K after 360 seconds, at least for one body position. In general, RF‐induced heating dominates the turbo spin‐echo sequence, whereas gradient‐induced heating prevails with EPI; the situation with fast imaging sequence with steady precession is more diversified. The RF effects are enhanced when the implant is within the RF coil, whereas the effects of gradient fields are maximized if the prosthesis is outside the imaging region. Cases for which temperature‐increase thresholds were exceeded were identified, together with the corresponding amount of tissue mass involved and the exposure time needed to reach these limits.

**Conclusion:**

The analysis confirms that risky situations may occur when a patient carrying a hip implant undergoes an MRI exam and that, in some cases, the gradient field heating may be significant. In general, exclusion criteria only based on whole‐body specific absorption rate may not be sufficient to ensure patients’ safety.

## 1 INTRODUCTION

The number of patients with a metallic implant undergoing a MRI procedure is increasing every year.^1^, ^2^, ^3^ Because metallic implants interact with the RF and gradient coil (GC) electromagnetic fields, this may produce a heating hazard. The MRI examinations proceed after assessing benefit and risk, the latter being based on theoretical and experimental evidence, as close as possible to clinical conditions.^4^, ^5^, ^6^

Dedicated standards cover implant safety in MRI, to provide guidelines for scanner manufacturers and health care professionals.^7^, ^8^, ^9^, ^10^ Additionally, several studies regarding implant safety in MRI are reported in the scientific literature. Most relate to heating associated with the exposure to RF fields associated with 1.5T and 3T systems,^11^, ^12^, ^13^, ^14^, ^15^, ^16^, ^17^, ^18^, ^19^, ^20^, ^21^ which usually leads to dissipated power close to elongated parts of the implants, giving rise to “hot spots” in the surrounding tissues; the spatial distribution and the maximum values of SAR10g (the specific absorption rate [SAR] averaged over 10 g of contiguous mass) depend on the body model, frequency, and relative position of coil and body.

Switched gradient‐field heating has been investigated to a lesser extent.^22^, ^23^, ^24^, ^25^, ^26^, ^27^, ^28^, ^29^, ^30^ Graf et al^22^ evaluated the heating of metallic objects, including a titanium hip prosthesis and an aluminum replica, during a 3D true fast imaging sequence with steady precession (TrueFISP) and concluded that, under specific conditions (eg, high duty cycle, long measuring time, metallic components with low resistance), gradient‐induced heating of conducting specimens could be expected. These general conclusions were in line with the results of experiments involving metallic components.^23^, ^24^

Zilberti et al^25^ suggested potential gradient‐induced heating in metallic hip implants and provided further evidence^26^ using an analytical model applied to a simplified metallic object.

Brühl et al^28^ investigated gradient‐switching heating involving the acetabular cup of a hip implant placed inside a gel phantom within a 3T scanner. The cup was placed orthogonally to the z‐axis in an off‐center position and exposed to an EPI sequence with continuous and trapezoidal z‐gradients. Gradient strength of 20 mT/m, slew rate of 200 T/(m s), and frequency of 2 kHz were set to the scanner limits when the sequence was run in “normal operating mode”. A temperature increase of 4 K was measured after 10 minutes. Arduino et al^29^ adopted a more complex approach, enabling the study of realistic clinical sequences. The computational procedure, validated by comparison with the experiments published in Brühl et al,^28^ led to a maximum temperature elevation exceeding 3 K computed using an EPI sequence. The effects of body position, frequency‐encoding direction, and thermoregulation were also investigated. Recently, Winter et al^31^ reviewed MRI‐related heating of implants and devices and presented concepts for risk assessment and quantification, and some first attempts toward an active safety management and risk mitigation.

Radiofrequency heating deposits energy directly in the biological tissues surrounding the implant, whereas GC fields deposit energy in the metallic implant. The spatial distributions of the two fields are significantly different. The RF B_1_ field is oriented primarily along the transverse plane and is as uniform as possible, whereas all three orthogonal components of the GC magnetic field contribute to the heating of metallic implants,^29^, ^30^ and the vector addition of the corresponding field distributions is required to predict the heating effects. Typical gradients range from 20 mT/m to 40 mT/m, with slew rates up to 200 T/(m s),^30^ and gradient field magnitude may reach several millitesla.^10^ Switched‐gradient trains also have a greater duty cycle than that of RF pulses within each TR.

An overview of the literature suggests that the significance of heating due to GC switching has been underestimated. This paper attempts to address this point by describing an in silico investigation into how the simultaneous exposure to GC and RF fields affects the temperature rise in patients with a metallic hip prosthesis during an MRI session. Novel features include consideration of realistic clinical sequences (in terms of signal waveforms, amplitudes, and repetitions), the superposition of the effects of the GC and RF fields, and the use of simulated temperature increase within the body, rather than SAR, as a safety metric.

## 2 METHODS

### 2.1 Anatomical body models and hip prosthesis

The study was performed using the “Duke” model (vs. 1.3) of a 34‐year‐old adult male, height = 1.77 m, weight = 70.3 kg (Virtual Population; Zurich MedTech AG, Zurich, Switzerland),^32^ consisting of 77 different biological tissues whose electrical and thermal properties were taken from the IT’IS database.^33^ Nonlinear thermal regulation effects for blood perfusion and metabolic heat were also included in the model.

A unilateral hip prosthesis was placed inside the Duke model (right side) in the appropriate anatomical position, taking care to avoid undesired overlapping between the tissues and the inserted object.

The prosthesis included a 142‐mm‐long stem, a 30‐mm‐diameter hemispherical head, an 8‐mm‐thick and 66‐mm‐diameter acetabular shell, a 34‐mm‐long screw, and a 10‐mm‐thick liner. The height, from the lower tip to the top of the femoral head, was 230 mm. The metallic components were CoCrMo alloy, a material used widely for prostheses components, and the liner was polyethylene. The properties of the implant materials are found in Table 1. CoCrMo is a conservative choice, as opposed to Ti‐alloy, as its electric conductivity results in a higher power deposition due to GC fields, whereas the RF heating is similar in both cases.

**TABLE 1:** Physical properties of the hip implant materials

	Electric conductivity	Relative permittivity	Relative permeability	Thermal conductivity	Specific heat capacity	Mass density
CoCrMo	1.16 MS/m	1	1	14 W/(m K)	450 J/(kg K)	8445 kg/m^3^
Polyethylene	0	2.25	1	0.47 W/(m K)	1900 J/(kg K)	940 kg/m^3^

Apart from thermoregulation effects, the electrical, magnetic, and thermal properties of both the biological tissues and the implant materials were assumed to be independent of temperature. The body model was segmented into cubic voxels with a resolution of 2 mm, generating a data set of about 8 millions of voxels.

### 2.2 Radiofrequency and gradient coils

The model of a generic high‐pass, 16‐rung, shielded circular birdcage coil model provided by a MRI system manufacturer was used to simulate the RF transmit coil (Supporting Information Figure S1). The coil was 713 mm in diameter and 450 mm in length. The end rings were 50 mm wide. All coil conductors were copper with conductivity of 59.5 MS/m. The cylindrical coil shield was 1500 mm long, 4 mm thick, with an inner diameter of 752 mm. Capacitors were connected across 1‐cm gaps in the end rings located between rungs, and loss was introduced at each capacitor by adding a 30.55‐kΩ parallel resistance. The coil was excited in quadrature using two ports placed across gaps in one of the end rings and was tuned to 123.2 MHz or 63.2 MHz when loaded with the body model, and impedance was matched by means of four additional capacitors inserted across gaps in that end ring.

The GCs represented a realistic setup used in MRI cylindrical bore scanners and consisted of three axial coils, one for each gradient, as shown in Supporting Information Figure S1. The overall length was 1.5 m, and the internal diameter was 720 mm. For each coil, a virtual model was generated, discretizing the conductors in filamentary elements, suitable for computing the magnetic flux density by Biot‐Savart law. The sensitivity constants of GCs were determined to correlate the current amplitude in each coil with the generated spatial gradient in the relevant direction inside the FOV region. These were 56.1 μT/(m A) for the X and Y coils, and 57.8 μT/(m A) for the Z coil.

### 2.3 Analyzed sequences

The pulse sequences analyzed were turbo spin‐echo (TSE), EPI, gradient‐echo (GRE), and TrueFISP. The parameters relevant to the TSE sequence, which were included in the analysis to emphasize RF heating effects, were similar to those proposed by ASTM.^9^ The EPI was designed to maximize the GC effects. The GRE and the TrueFISP parameters were selected to be representative of typical MRI sequences. In particular, the GRE represents a low‐impact, multislice $T_{2}^{*}$‐weighted sequence, whereas the TrueFISP was chosen to investigate the thermal effects of an extremely fast pulse sequence often deployed in clinical practice.^34^ The sequences were designed with the same set of parameters for both 1.5T and 3T simulations.

Whole‐body SAR (SARwb) values were calculated for different positions of Duke relative to the birdcage coil, for all sequences and for both 1.5T and 3T cases (see Supporting Information Table S1). SARwb was below 2 W/kg in all cases except for the TSE sequence, in which case the limit was originally exceeded for all positions at 3 T (range 3.19 W/kg‐7.43 W/kg) and for the position of thorax imaging at 1.5 T (2.29 W/kg). To make the TSE sequence SARwb‐compliant, a dead time was introduced after each TSE acquisition to reduce the maximum time‐averaged SARwb to 2 W/kg. This was chosen as constant for all positions (based on the worst case) or variable for each imaging position. For the 3T cases, the dead time ranged from 2.41 seconds to 5.67 seconds, the latter resulting in 47 TSE acquisitions (each with eight repetitions) within about 360 seconds, rather than the original 176 TSE acquisitions. For the 1.5T cases, it was 0.3 seconds, resulting in 151 acquisitions within the same time interval.

Regardless of the actual sequence duration, the thermal effects were mostly evaluated after 360 seconds, even if some analyses were extended to 900 seconds. Therefore, sequences were repeated until they reached either 360 seconds or 900 seconds. Table 2 summarizes the parameters associated with the sequences described previously (sequence waveforms are reported in Supporting Information Figures S2‐S5).

**TABLE 2:** List of the most important parameters associated with the four analyzed sequences

	TSE	EPI	GRE	TrueFISP
Flip angle (degrees)	90/180	90	20	45
RF pulse duration (ms)	1.6/1.6	1.6	10	1
RF time/bandwidth product	4/4	4	10	4
TE (ms)	6	21	20	3.2
TR (ms)	260	43	500	6.4
Matrix	128 × 128	64 × 64	256 × 256	256 × 256
FOV (mm × mm)	450 × 450	182 × 182	140 × 140	120 × 180
Slice thickness (mm)	10	8	4	4
Readout bandwidth (kHz)	69	150	20.83	126.3
Slices per TR	2	1	11	1
Echo train length	16	—	—	—
Maximum gradient slew rate (T/m/s)	180	167	130	200
Dead time (seconds)^a^	0 ÷ 5.67	0	0	0

Note

An apodization factor equal to 0.5 has been used for all of the sinc‐shaped RF pulses.

^a^ A dead time is introduced in the TSE sequence after each single acquisition (eight times the TR), to limit the whole‐body specific absorption rate (SARwb) to 2 W/kg. The values of the dead time depend on the body position within the scanner.

To investigate possible RF and GC heating variation associated with a specific sequence, two additional variations of the TrueFISP sequence were considered. The modified parameters were chosen to maintain the same intrinsic image “weighting” FOV and resolution. In the first TrueFISP variation (TrueFISPv1), the RF pulse duration was increased from 1 ms to 1.5 ms, keeping the same RF time‐bandwidth product. In the second TrueFISP variation (TrueFISPv2), the readout bandwidth was extended to 200 kHz, keeping the RF pulse duration to 1 ms. All other sequence parameters were unchanged.

### 2.4 Computation of power deposition

#### 2.4.1 Radiofrequency simulations

The RF coil structure and the implanted Duke model (2‐mm resolution) were imported into a commercial finite‐difference time domain solver (*SEMCAD X* v14.8.6; Schmid & Partner Engineering, Zurich, Switzerland).^35^ Approximately 4.4 × 10^7^ voxels were created on a variable grid within the *SEMCAD* environment. Minimum grid steps ranged from about 1 mm to 50 mm in free space away from structures. Medium‐strength (>95% absorption) uniaxial perfectly matched layers absorbing boundary conditions were set at the faces of the computational domain. A strict convergence parameter (−75 dB) was set. Simulations were performed on PCs with Intel Core i7‐3820 3.6 GHz CPUs, 16 GB RAM, and NVIDIA Tesla C2075 GPU cards (Santa Clara, CA). Initially, a broadband simulation was performed to obtain frequency‐dependent S‐parameters, and then a harmonic simulation was performed at the appropriate resonant frequency to obtain spatial dependencies of the electric and magnetic fields and SAR. A typical run time for a harmonic simulation was 13 hours. The field maps of power density *P*_RF_ (computed from the electrical field map) and B_1_ were remapped to an isotropic 2‐mm grid, such that local volume integrals were conserved. Maps of SAR (*P*_RF_/ρ) are determined by combining *P*_RF_ with the distribution of mass density ρ in the biological tissues.

#### 2.4.2 Gradient coil simulations

Following the approach described in Arduino et al,^29^ the GC simulations were restricted to the region of the metallic implant. This numerical approach is based on time‐harmonic electromagnetic solutions. For each GC sequence, a representative time interval Δ, either the time frame including a whole‐slice acquisition for single‐shot (eg, for the EPI sequence), or the TR for non‐single‐shot sequences, was chosen. The signal restricted to the interval Δ was divided into subsignals, which were either periodic or aperiodic. Each subsignal was then represented through a truncated Fourier expansion through fast Fourier transform. The electromagnetic problem was solved for each harmonic component of each single subsignal using a hybrid finite element/boundary element solver, as described in Bottauscio et al.^36^ Thus, a set of solutions, each one providing the induced currents in each voxel of the implant for a given harmonic, was generated. The harmonic decomposition of the induced current was then moved back to the time domain for each subsignal, and superimposed coil by coil to reconstruct the actual time waveform of the local induced current density vector. Finally, the instantaneous power density (*P*_GC_) and the energy density in each voxel during each time step were computed. The computation of *P*_GC_ was repeated for each successive time interval. Simulations were performed on Intel Xeon CPU E5‐2680 v2, 128 GB RAM, with NVIDIA K80 GPU card.

#### 2.4.3 Combining RF and GC power

Values of *P*_RF_ and *P*_GC_, previously computed in each voxel, were combined to determine the total power density *P*_TOT_. The value of *P*_GC_ was zero in the tissues outside the implant, whereas *P*_RF_ was zero inside the implant (due to perfect electric conductor, conditions^37^). In each voxel, the power was averaged over the time step of the marching method, inserting, when required, idle times between two successive intervals Δ.

Depending on the value assigned to the volume power density (*P*_em_) in the thermal problem (see Equation 1 in section 2.5), for each sequence, three thermal solutions can be obtained: P1, in which the effects of only the GC are considered (ie, *P*_em_ = *P*_GC_); P2, when only the RF coil is active (ie, *P*_em_ = *P*_RF_); and P3, which combines the effects of both sets of coils (ie, *P*_em_ = *P*_TOT_). This enables the global heating to be determined according to the contributions of each single power source. Because the bioheat model adopted in this work accounts for thermoregulation effects in a nonlinear fashion (see section 2.5), the temperature increase computed in P3 may not be simply the sum of the increases obtained by applying P1 and P2.

### 2.5 Thermal modeling

Thermal simulations were carried out by solving a form of Pennes’ bioheat equation,^38^ expressed in terms of temperature elevation Δ*T* with respect to the temperature at rest *T*_0_.^39^ This avoids knowing the spatial distribution of *T*_0_, which is determined by the metabolic heating, the diffusion‐perfusion phenomena, the blood temperature *T*_b_, and the thermal boundary conditions at the skin.

Thermoregulation processes, affecting blood perfusion and metabolic heating, were considered as proposed in Laakso and Hirata.^40^ The temperature‐dependent perfusion coefficient is *h*_b_ = *L*_B_ *h*_b0_, where *L*_B_ = $2^{\left(T-T_{0}\right)/\Delta B}$ is the local temperature‐dependent multiplier (for *T* > *T*_0_); and *h*_b0_ is the perfusion coefficient at *T*_0_. Coefficient Δ*B* is set to 1.6 K, and the value of *L*_B_ was limited to 15 for all tissues except for skin, in which case the limit was 32. Metabolic heat production was assumed to be dependent on local tissue temperature through a factor *L*_met_ = $1.1^{\left(T-T_{0}\right)}$, as in Laakso and Hirata.^40^ This thermoregulation model assumes an instantaneous regulation, disregarding the response times of the thermoregulatory process that regulates the core body temperature and is appropriate for the local temperature increase.

The resulting bioheat equation is

(1) $$\rho c_{p}\frac{\partial \left(\Delta T\right)}{\partial t}=\nabla \cdot \left[\lambda \nabla \left(\Delta T\right)\right]‐2^{\Delta T/\Delta B}h_{b0}\Delta T+\left(1.1^{\Delta T}‐1\right)P_{\text{met}0}+P_{\text{em}},$$

where *ρc*_p_ is the volumetric heat capacity; *λ* is the thermal conductivity; *P*_met0_ is the volume power density associated with the metabolic process evaluated at rest; and *P*_em_ is the volume power density deposited by the applied RF and/or GC fields. Robin boundary conditions ${\left(\lambda \partial \left(\Delta T\right)/\partial n\right|}_{\partial V}=‐h_{\text{amb}}\Delta T$ were applied at the body surface (∂V), with a heat exchange coefficient *h*_amb_ = 7 W/(m^2^ K). Other nonlinear contributions due to thermoregulatory processes, like sweating, which would affect the heat exchange coefficient *h*_amb_,^41^ were neglected.

Equation 1 was solved numerically by a finite‐difference method using a Douglas–Gunn time‐split scheme. The characteristics of the Douglas–Gunn algorithm allow a parallel implementation on GPUs, leading to an efficient solution on the entire voxelized body model. Details of the numerical implementation can be found in Arduino et al.^39^

### 2.6 Test conditions

**FIGURE 1:**
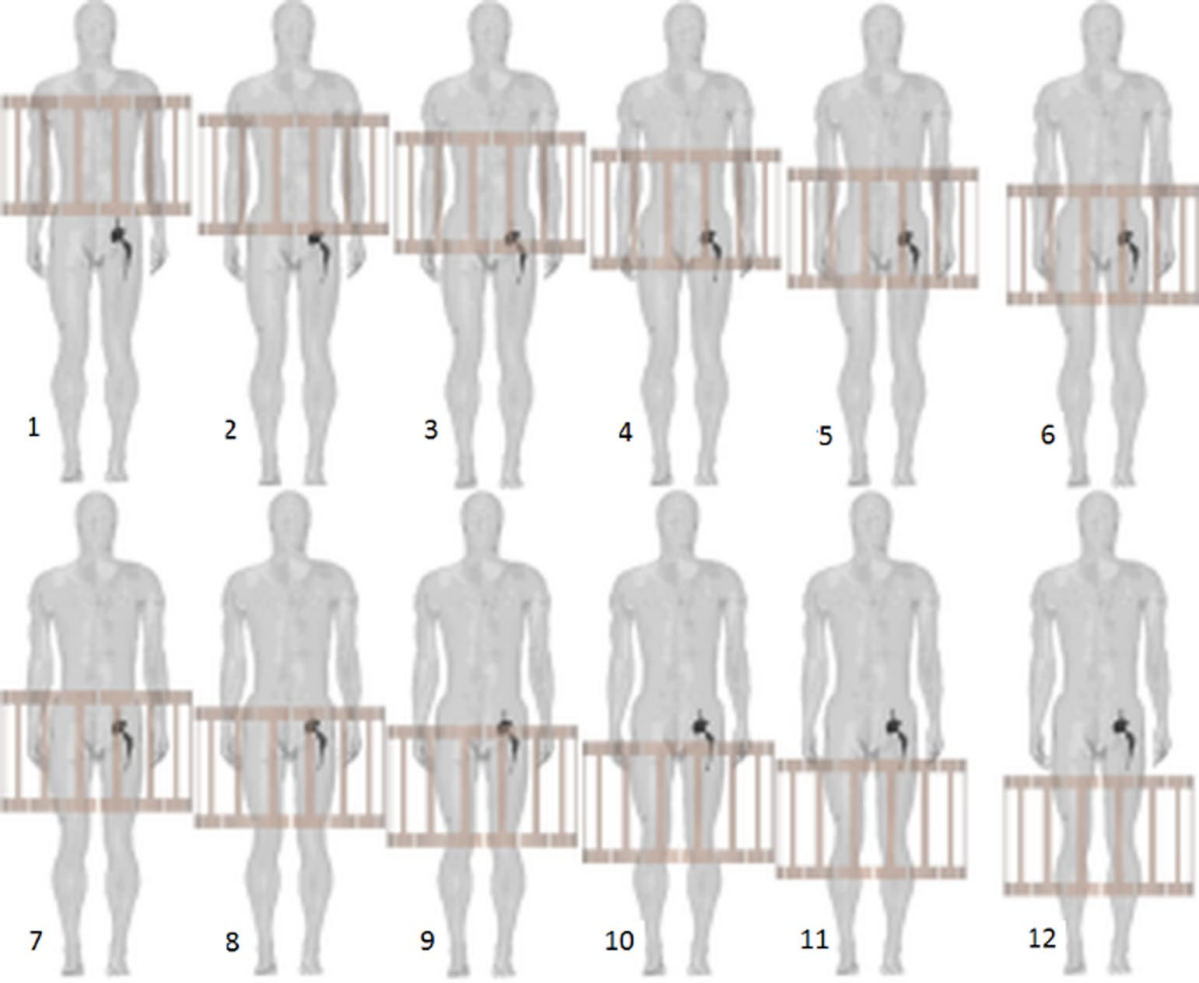
Human body model positions with right‐side hip prosthesis. A back view of the human body is reported. The body positions are numerated from 1 (thorax MRI) to 12 (femur/knee MRI), which correspond to the following positions of the implant head along the z‐axis with respect to the isocenter: +288 mm, +224 mm, +160 mm, +96 mm, +32 mm, −32 mm, −96 mm, −160 mm, −224 mm, −288 mm, −352 mm, and −416 mm

Simulations were performed for 1.5T and 3T conditions. For each case, Duke was moved in increments of 64 mm along the scanner’s longitudinal axis, to explore different relative positions between implant and coils. Figure 1 shows the 12 positions investigated, including cases in which the implant is either entirely inside or outside the footprint of the RF body coil. Each position investigated is associated with one of four generic imaging zones—femur/knee, pelvis, abdomen, and thorax—relevant to MRI procedures facilitating interpretation of the results from a clinical viewpoint.

A total of 288 simulations were performed, accounting for all combinations of the four selected sequences, 12 body positions, three power cases (P1, P2, and P3), and two Larmor frequency values.

### 2.7 Rationales for temperature increase evaluation

IEC 60601‐2‐33^8^ limits local temperature to 39 °C for normal operation mode. ICNIRP^43^ considers 41 °C and above as potentially harmful and recommends a maximum temperature increase of 2 K for all tissues in the upper arm, forearm, hand, thigh, leg, foot, pinna and the cornea, anterior chamber and iris of the eye, epidermal, dermal, fat, muscle, and bone tissue. For all tissues in the head, eye, abdomen, back, thorax, and pelvis, excluding those referred to previously, the maximum increase is 5 K.

Preliminary simulations considering the thermal equilibrium of the body model (not reported here) suggested that a conservative approach assume 38 °C as the maximum local temperature within the body model before exposure. This results from considering the variability of several parameters (including *T*_b_, kept constant within each simulation but varied in the range of 37 °C‐37.5 °C), and from observing that the minimum temperature at rest occurred in the skin and the maximum temperature, approximately 0.6 K higher than blood temperature, was always in the heart. For tissues close to the hip prosthesis, the average and maximum temperatures at rest were 0.2 K lower and 0.3 K larger than the blood temperature, respectively.

Therefore, thresholds for temperature increase equal to 1 K and 3 K, corresponding to the temperature limits of 39 °C and 41 °C set in Refs 8, 42 and 43, were used to assess the results of the thermal simulations. More details about the rationale behind this choice are reported in Supporting Information Section S5.

A region of influence was introduced to limit the analysis to a volume in which the presence of the metallic implant sensibly modifies the temperature increase, accounting for both GC and RF heating. A parallelepiped box of size 21.7 × 18.8 × 28.2 cm^3^, including the bounding box of the implant, was determined according to the procedure based on SAR and temperature‐increase variations caused by the metallic implant described in Supporting information Section S4.

## 3 RESULTS

**FIGURE 2:**
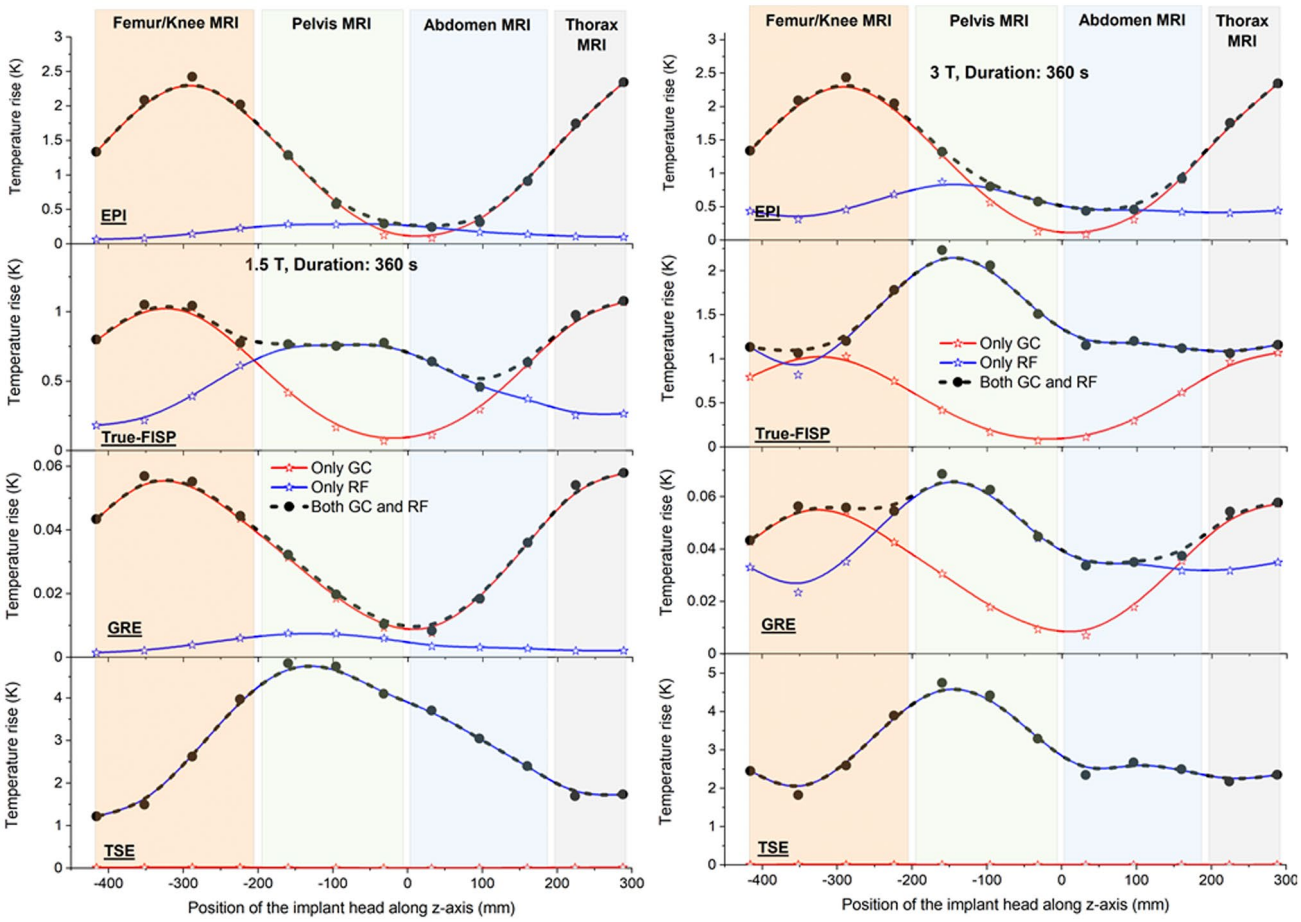
Maximum temperature increase after 360 seconds of exposure versus the axial position of the hip implant in the coils; z = 0 corresponds to the isocenter. Points in the plots correspond to the 12 body positions shown in Figure 1 and numerated from 1 to 12. Results for EPI, true fast imaging sequence with steady precession (TrueFISP), gradient‐echo (GRE), and turbo spin‐echo (TSE) (with constant dead time) sequences are shown, considering the effects of the RF coil alone, the GC alone, and both coils together. Symbols denote the computed values, and the interpolating lines show the trends. The four imaging regions (femur/knee, pelvis, abdomen, and thorax) related to the axial position are also indicated. Left side: Results for 1.5 T. Right side: Results for 3 T. Note that the overall maximum is plotted. This may differ only slightly from that associated with a single source, because of the different spatial distributions of temperature increase within the body associated with each source. Abbreviation: GC, gradient coil.

Figure 2 shows the maximum temperature increase after 360 seconds versus the position of the hip implant along the z‐axis of the scanner. For different sources (GC, RF, and both), the position of the maximum is dependent on how the two fields produce heating (in the metal, in the tissues, or both). When considering a given position, the effects from the sources are not simply additive, because of the nonlinear thermoregulation effects. In addition, when considering the overall maximum, the result may differ only slightly from that associated with a single source, because of the different spatial distributions of temperature increase. The maximum temperature increase occurs for thorax or femur/knee imaging for the GC sources (when the implant is outside the imaging region) and for pelvis imaging for the RF source contribution (when the implant is inside the RF coil). Smaller changes are found for abdomen imaging for both GC and RF sources.

The TSE sequence (with SARwb = 2 W/kg for the worst‐case body/coil positioning [see Supporting Information Table S1] for both 1.5T and 3T cases) is the most aggressive one, and the predominant heating is caused by RF, whereas for EPI, GC fields are the dominant sources. The contributions of the two fields are comparable for TrueFISP and GRE sequences. The GRE sequence is the least aggressive, both in terms of RF and GC contributions, and is not further investigated.

The global heating effect caused by RF is slightly higher at 3 T than at 1.5 T, whereas the GC contribution is independent of the static field intensity. Comparison of RF heating with and without the implant is shown in Supporting Information Figure S7.

Table 3 lists temperature increases after 360 seconds at the positions associated with the worst case for each combination of imaging region and sequence shown in Figure 2. To make direct comparisons of temperature increases for the TSE sequence in this table, the dead times applied after each acquisition used to reduce the whole‐body SAR to 2 W/kg depend on the body/coil position. For thorax MRI (z = +228 mm), abdomen MRI (z = +96 mm), pelvis MRI (z = −160 mm), and femur/knee MRI (z = −224 mm), the dead times for 3 T are 5.67 seconds, 4.46 seconds, 3.25 seconds and 2.41 seconds, respectively. The temperature increases after a longer exposure time (900 seconds) are also reported when both GC and RF fields are applied. In some cases, the maximum Δ*T* is not determined directly by the presence of the implant (eg, for thorax and abdomen MRI at 3 T). The analysis was deepened in each of the four imaging regions for the worst‐case exposure (Figure 2), referring to the region of influence as defined in section 2.7.

**TABLE 3:** Max temperature increase Δ*T* in the human body (after 360 seconds) for each imaging region and sequence at 1.5 T and 3 T

MRI region		FISP	EPI	TSE with unique DT	TSE with DT variable with position	GRE
**1.5 T**						
Thorax	DT (seconds)	0	0	0.30	0.30	0
	GC	**1.07**	2.34	0.020	0.020	**0.058**
	RF	**0.27**	0.10	1.73	1.73	**0.002**
	Both	**1.08/1.60**	2.34/3.16	1.73/2.20	1.73/2.20	**0.058/0.094**
Abdomen	DT (seconds)	0	0	0.30	0	0
	GC	0.11	0.90	<0.001	<0.001	0.036
	RF	0.64	0.14	3.71	4.16	0.003
	Both	0.64/0.69	0.91/1.29	3.71/3.84	4.16/4.56	0.036/0.056
Pelvis	DT (seconds)	0	0	0.30	0	0
	GC	0.07	1.27	**0.012**	**0.013**	0.031
	RF	0.78	0.29	**4.82**	**5.34**	0.008
	Both	0.78/0.84	1.29/1.79	**4.82/5.01**	**5.34/5.79**	0.032/0.050
Femur/knee	DT (seconds)	0	0	0.30	0	0
	GC	1.04	**2.42**	0.018	0.020	0.056
	RF	0.22	**0.15**	3.97	4.44	0.002
	Both	1.05/1.57	**2.42/3.26**	3.97/4.50	4.44/4.96	0.056/0.091
**3 T**						
Thorax	DT (seconds)	0	0	5.67	5.67	0
	GC	1.07	2.34	0.006	0.006	0.057
	RF	1.16	0.44	2.35	2.35	0.035
	Both	1.16/1.74	2.35/3.17	2.35/3.04	2.35 (2.59)/3.04	0.058/0.095
Abdomen	DT (seconds)	0	0	5.67	4.46	0
	GC	0.30	0.90	<0.001	<0.001	0.035
	RF	1.20	0.42	2.67	2.84	0.032
	Both	1.20/1.76	0.92/1.32	2.67/3.37	2.84 (2.94)/3.78	0.037/0.060
Pelvis	DT (seconds)	0	0	5.67	3.25	0
	GC	**0.42**	1.27	**0.004**	**0.006**	**0.031**
	RF	**2.23**	0.87	**4.75**	**6.06**	**0.068**
	Both	**2.24/2.62**	1.33/1.86	**4.75/5.13**	**6.06 (2.13)/6.26**	**0.068/0.083**
Femur/knee	DT (seconds)	0	0	5.67	2.41	0
	GC	0.75	**2.42**	0.006	0.01	0.055
	RF	1.78	**0.46**	3.89	5.44	0.035
	Both	1.78/2.13	**2.44/3.28**	3.89/4.32	5.44 (1.94)/5.94	0.056/0.094

Note

For the case of both GC and RF field applied, the max temperature increase Δ*T* after 900 seconds is also reported after the slash (/) symbol. For the TSE sequence, the maximum Δ*T* values are reported for imposing a unique dead time (DT) for all positions (0.30 seconds for 1.5 T and 5.67 seconds for 3 T), which limits the SARwb to 2 W/kg in the worst condition and adjusting the DT to limit the SARwb to 2 W/kg for each body position. For the TSE sequence with variable DT at 3 T, the maximum Δ*T* obtained after 360 seconds without the presence of the implant is reported in round brackets, showing how these maximum Δ*T* values are not always determined by the presence of the implant (eg, for thorax and abdomen MRI). The maximum heating reached by each sequence for 1.5 T and for 3 T with both sources active is reported in bold.

**FIGURE 3:**
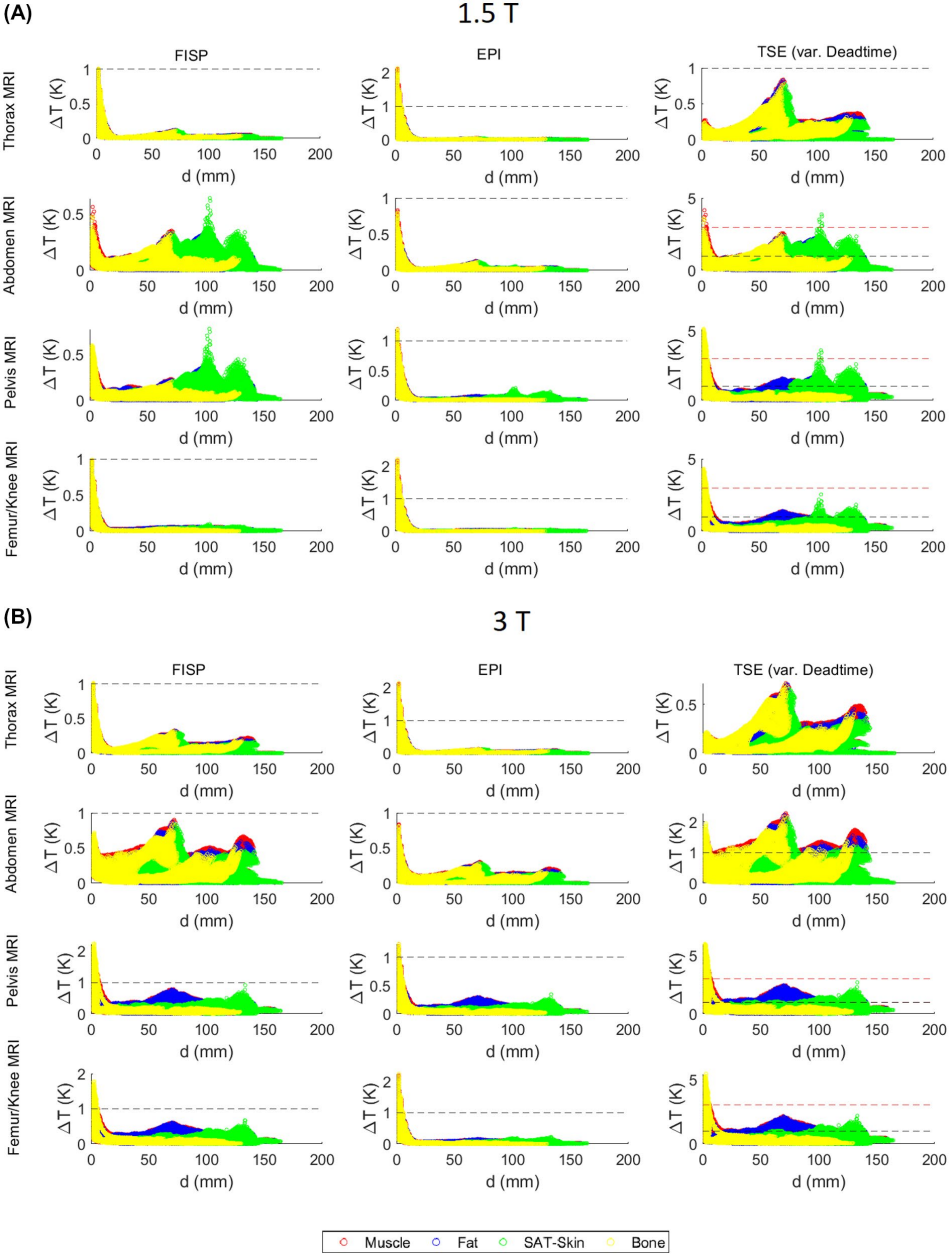
Plots of the temperature increase Δ*T* (after 360 seconds) of each voxel belonging to the region of influence versus the minimum distance *d* from the implant surface. Only the tissues most affected by the heating are plotted (bone, fat, muscle, and subcutaneous adipose tissue/skin). The considered Δ*T* thresholds of 1 K and 3 K are indicated by a black and red dashed line, respectively. Additional data for more tissues are reported in the Supporting Information. The worst conditions, in terms of maximum heating, within the four imaging regions (femur/knee, pelvis, abdomen, and thorax) are considered (see also Table 2). A, Results at 1.5 T. B, Results at 3 T. Because the appearance of the plots depends on the order in which the contributions from different tissues are added (ie, legend order: “muscle” first and “bone” last), for cases in which the same temperature/distance occurs in more than one tissue, earlier points are overwritten

In Figure 3, for each voxel belonging to the region of influence and made of bone, fat, muscle, subcutaneous adipose tissue or skin, the temperature increase after 360 seconds in the presence of both field sources (RF and GC) is plotted against its distance to the implant. The results are shown for the TrueFISP, EPI, and TSE sequences at 1.5 T and 3 T. Similar plots, involving more tissues, are shown in Supporting Information Figures S8‐S11. Because the appearance of the plots depends on the order in which the contributions from different tissues are added, there are cases in which the same temperature/distance occurs in more than one tissue and earlier points are overwritten. For abdomen MRI at 1.5 T, only exposure to TSE results in a temperature increase greater than 1 K. For thorax MRI, this happens for all reported tissues when the TSE sequence is applied. With the EPI and the TrueFISP sequences, the temperature increases in subcutaneous adipose tissue/skin are lower.

For pelvis MRI and TSE, connective tissue also crosses the threshold of 1 K. With EPI, this happens only for bone, muscle and fat, whereas only bone and muscle are over 1 K with TrueFISP at 3 T. Finally, for femur/knee MRI, the bone, fat, and muscle overcome the threshold of 1 K with all of the sequences, with the exception of TrueFISP at 1.5 T.

When GC effects are more relevant, the voxels exceeding the threshold are primarily close to the implant; this is not always true when the RF field dominates the heating.

**FIGURE 4:**
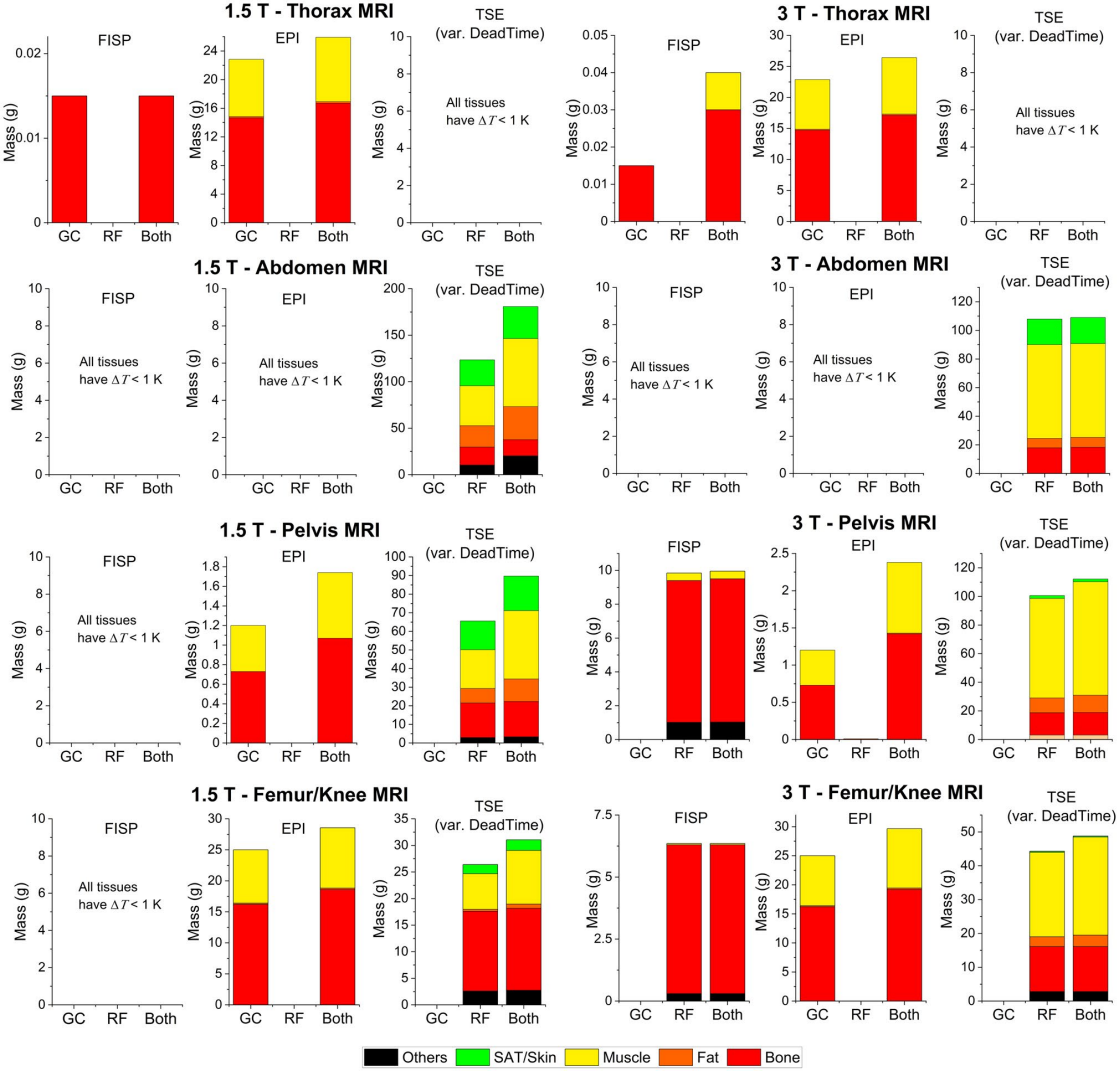
Mass of tissues around the implant that exhibit a temperature increase Δ*T* (after 360 seconds) greater that the threshold of 1 K. From top to bottom, the group of results for 1.5 T (thorax MRI, abdomen MRI, pelvis MRI, and femur/knee MRI) and the group of results for 3 T. For the TSE sequence, only the masses computed with variable dead times for all positions are reported. Within each bar, the color indicates the fraction associated with a given tissue

The histograms in Figure 4 show the mass of tissue within the region of influence, which undergoes a temperature increase Δ*T* > 1 K. The contribution from each tissue to the total mass is highlighted using color codes. Differences due to the selected sequence, the imaging region, the power case, and the Larmor frequency are shown.

The involved mass is greater with TSE; the extent of the RF heating results in a temperature increase of significantly large portions of tissue with Δ*T* > 1 K (up to 110 g at 3 T and up to 175 g at 1.5 T). With EPI, the greatest heated mass is about 30 g, found in the thorax and femur/knee MRI. The greatest mass for TrueFISP (~10 g) is found for the pelvis MRI at 3 T.

Only the TSE sequence produces Δ*T* > 3 K. At 1.5 T (variable dead time), masses of bone (up to about 6 g) and muscle (less than 0.1 g) are heated above this threshold in the abdomen, pelvis, or femur/knee MRI. At 3 T (variable dead time), masses of bone (from 3 g to about 6 g) are heated above this threshold with pelvis or femur/knee MRI.

**FIGURE 5:**
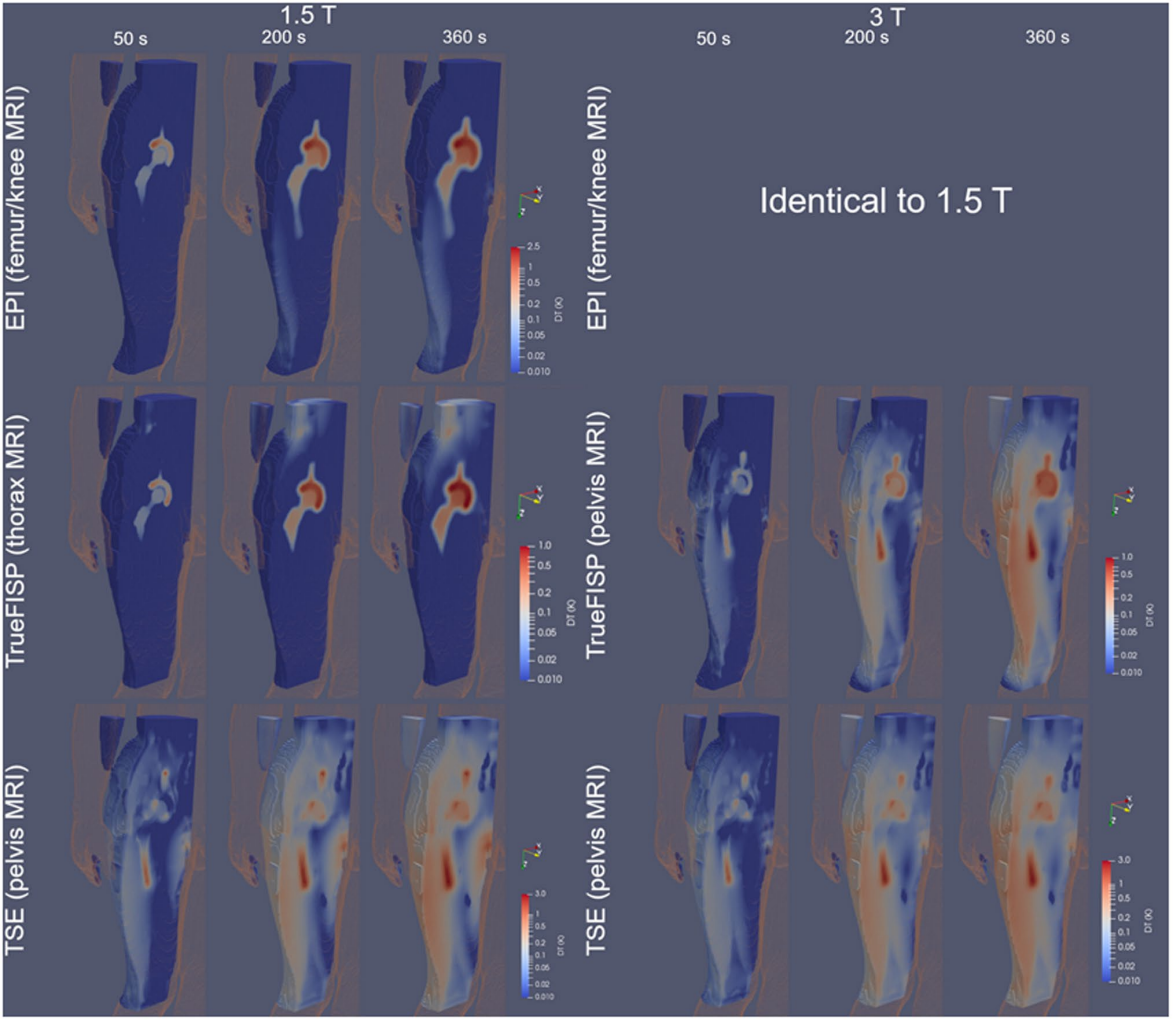
Spatial evolution, at different time instants, of the temperature increase around the prosthesis for TSE, FISP, and EPI sequences (screenshots after 50 seconds, 200 seconds, and 360 seconds). The positions where the maximum temperature increase is found are considered (see Figure 2), separately, for TSE, FISP, and EPI at 1.5 T (left group of plots) and 3 T (right group of plots). For the 3T group, the results for the EPI sequence (for which GC‐induced heating prevails) are not reported, being almost identical to those at 1.5 T. The results for the TSE sequence refer to a constant dead time of 5.67 seconds

Figure 5 shows the thermal evolution around the implant in terms of the distribution of the temperature increase after 50 seconds, 200 seconds, and 360 seconds from the beginning of the exposure. Only the imaging regions associated with the maximum temperature increases are considered.

Table 4 lists the variation of the maximum temperature increase for two variants of the TrueFISP sequence, as described in section 2.3. By increasing the RF pulse duration (TrueFISPv1), Δ*T* varies from 1.08 K to 1.33 K at 1.5 T and from 2.24 K (resp. 1.16 K) to 1.53 K (resp. 1.34 K) for pelvis MRI (resp. thorax MRI) at 3 T. By increasing the readout bandwidth (TrueFISPv2), the temperature increase remains equal to 1.08 K at 1.5 T and to 2.24 K at 3 T (pelvis MRI), whereas it varies from 1.16 K to 1.34 K for thorax MRI at 3 T.

**TABLE 4:** Maximum temperature increase Δ*T* (after 360 seconds) for three versions of the TrueFISP sequence and the positions where the maximum Δ*T* is found

MRI region	Body position	TrueFISP			TrueFISPvs1			TrueFISPvs2		
		GC	RF	Both	GC	RF	Both	GC	RF	Both
1.5 T										
Thorax	1	1.07	0.27	1.08	1.32	0.178	1.33	1.32	0.27	1.33
3 T										
Pelvis	8	0.42	2.23	2.24	0.44	1.53	1.53	0.47	2.24	2.24
Thorax	1	1.07	1.16	1.16	1.32	0.79	1.34	1.33	1.16	1.34

Note

Body positions refer to those defined in Figure 1.

The times taken to exceed the temperature‐increase thresholds (over the entire body) are given in Table 5. In most cases, exposures of up to 15 minutes resulted in Δ*T* > 3 K; exceptions were TrueFISP (for all imaging positions and for 1.5 T and 3 T), EPI (for abdomen and pelvis for 1.5 T and 3 T), and TSE for thorax at 1.5 T. The shortest time to exceed the limit (29 seconds) was found for TSE pelvis imaging at 3 T. It is worth noting that for pelvis and femur/knee imaging with TSE at 3 T, the maximum temperature increase occurs in the arm and elbow, independently of the presence of the implant. At 1.5 T, Δ*T* exceeds 1 K for TSE after 9‐14 seconds for abdomen, pelvis, and femur/knee imaging, and after 148 seconds for thorax imaging. For EPI and femur/knee imaging, the threshold was exceeded after 60 seconds. At 3 T, the times taken for Δ*T* to exceed 1 K for TSE ranged from 6‐28 seconds for abdomen, pelvis, and femur/knee imaging, to 102 seconds for thorax imaging. In the case of EPI at 3 T and femur/knee and thorax imaging, the threshold was exceeded after 66‐82 seconds.

**TABLE 5:** Times (in seconds) from the beginning of the sequence when the thresholds on Δ*T* (1 K or 3 K) are exceeded due to the combined effect of RF and GC fields

MRI region	1.5 T								3 T							
	FISP		EPI		TSE with unique DT		TSE with variable DT		FISP		EPI		TSE with unique DT		TSE with variable DT	
	> 1 K	> 3 K	> 1 K	> 3 K	> 1 K	> 3 K	> 1 K	> 3 K	> 1 K	> 3 K	> 1 K	> 3 K	> 1 K	> 3 K	> 1 K	> 3 K
Thorax	313	—	66	735	148	—	148	—	286	—	66	730	102	769	102	769
Abdomen	—	—	449	—	10	93	9	64	240	—	432	—	33	513	28	316
Pelvis	—	—	209	—	12	83	10	64	41	—	198	—	9	64	6	29
Femur/																
knee	328	—	60	658	16	148	14	105	69	—	82	641	9	126	6	37

Note

If the threshold on temperature increase is not reached within the maximum considered exposure time (900 seconds), no time value is shown. For the TSE sequence, the times are reported for the case with a single DT for all positions (0.30 seconds for 1.5 T and 5.67 seconds for 3 T), which limits the SARwb to 2 W/kg in the worst condition, and for the case with DT adjusted to limit the SARwb to 2 W/kg for each body position.

## 4 DISCUSSION

Numerical simulations of heating of a unilateral CoCrMo hip prosthesis within an anatomically realistic body model, due to the electromagnetic fields from a RF transmit coil, and a set of GCs representative of those used in 1.5 T and 3 T cylindrical bore scanners are described. Twelve axial positions of the body and representatives of the femur/knee, pelvis, abdomen, and thorax imaging are considered, and signals simulating TSE, EPI, GRE, and TrueFISP sequences are used to drive the coils. The results are discussed in terms of temperature increase within the body, rather than SAR, as the RF safety metric. Considering the limit of 39 °C for the local temperature under normal operation mode,^8^ and the value of 41 °C as potentially harmful,^43^ thresholds for temperature increase equal to 1 K and 3 K were used to assess the results.

For sequences with a high RF contribution (eg, TSE), tissue heating due to the presence of the prosthesis is dependent on the axial position of the prosthesis within the coils; the maximum occurs when the prosthesis is positioned approximately centrally within the footprint of the RF coil (see Figures 1 and 2)—a position representative of pelvis imaging. This agrees with a previous study.^14^ In the present study, for both the 1.5T and 3T cases, the TSE sequence produced the largest temperature increases for all imaging regions investigated (ie, approximately 5.3 K to 6.1 K for 1.5 T and 3 T, respectively). The ratios of maximum temperature increases predicted for the 3T and 1.5T cases were in the approximate range of 0.7 (for abdomen imaging) to 1.4 (for thorax imaging). All of these results suggest that SARwb does not represent a reliable metric for predicting heating in the presence of implants.

The thresholds for temperature increase Δ*T* > 1 K and Δ*T* > 3 K were exceeded for most sequences at both 1.5 T and 3 T. Exceptions were for the TrueFISP sequence for abdomen and pelvis imaging at 1.5 T, and the GRE sequence for all positions. Thresholds were exceeded in the shortest times for TSE, which ranged from 6 seconds (at 3 T for pelvis and femur/knee imaging) to 9 seconds (at 1.5 T for abdomen imaging) for Δ*T* > 1 K. In these cases, Δ*T* > 3 K was reached after times in the range of 29 seconds to 64 seconds.

In the case of less aggressive sequences, such EPI for femur/knee and thorax imaging, Δ*T* > 1 K was reached after 60‐82 seconds (similarly for 1.5 T and 3 T). A similar but slightly smaller increase in temperature was predicted for a thorax imaging position. In these positions, the prosthesis was outside the footprint of the transmit coil, and the GC fields contributed most to tissue heating. Because contributions of the RF fields were small compared with GC fields, predicted temperature increases for both the 1.5T and 3T cases were similar. Although femur/knee imaging and EPI would not be a frequently used combination, the result is nonetheless interesting, because in our study, EPI is representative of any sequence whose heating contribution is dominated by the GCs.

For the TrueFISP sequence at 1.5 T, GC fields mostly contributed to heating for body positions relevant to thorax and femur/knee imaging; larger temperature increases were predicted in these cases compared with pelvis and abdomen imaging, for which RF fields contributed the most. However, at 3 T, the contribution from the RF fields increased and exceeded the GC contributions; the maximum increase in temperature was observed for the pelvis imaging position. The GRE sequence produced low heating for all imaging positions.

In general, the simultaneous occurrence of RF and GC thermal heating does not sensibly modify the maximum temperature increase due to the prevailing phenomenon, because of the different spatial distribution of the two thermal effects.

Methods for reducing the heating induced by the interaction between RF fields and metallic implants have been proposed.^44^, ^45^, ^46^, ^47^ These include using an elliptical rather than circular polarization of the B_1_ field,^45^ parallel transmission techniques,^46^ or a cloaking strategy.^47^ Other options to reduce temperature increase due to both RF and GC fields include changing the deployed sequence when possible, tuning the RF pulse parameters (see, for example, the effects in the case of TrueFISP reported in Table 4), and varying the frequency encoding direction (especially for EPI^29^).

The results presented predict the heating associated with a prosthesis in a patient during several potential MR procedures and not simply the heating propensity of the prosthesis determined by methods described in ASTM F2182‐19.^9^ These results emphasize the fact that compliance with standard test methods does not address all safety concerns,^9^ and standard test methods should be complemented by realistic simulations such as those described previously. In particular, in contrast to exposure within a phantom, the implant is realistically positioned and orientated within a multitissue environment with temperature‐dependent perfusion and metabolism, both important factors in determining temperature increase.

An exposure duration of up to 15 minutes is recommended by the ASTM.^9^ Predictions of realistic simulations, such as those reported here, suggest that recommended temperature limits can be exceeded even after much shorter exposures. Often, longer exposures are associated with clinical protocols that involve a combination of sequences. In these cases, temperature increases will be influenced by the order of execution of the different sequences and by possible dead times properly distributed between consecutive sequences. This simply translates in a scheduling problem, which is addressed today on the basis of parameters that do not account for the presence of a prosthesis.^48^ The study of possible criteria to define prosthesis safety scheduling protocols can be the subject of future work.

## 5 CONCLUSIONS

Simulations of the temperature increase due to interactions of RF and GC fields with an adult male body model implanted with a unilateral CoCrMo hip prosthesis are described. Realistic clinical sequences (TSE, EPI, GRE, and TrueFISP) and the superposition of the spatially different effects of the GC and RF fields are investigated for imaging thorax, abdomen, pelvis, and femur/knee regions with 1.5T and 3T systems. Results from this kind of numerical study complement phantom‐based measurements of implant heating that are compliant with standard test methods.

The RF fields dominate heating during TSE sequences, regardless of relative body/RF coil positioning. Attention is drawn to the significance of the contribution of GC fields in sequences such as EPI and TrueFISP when imaging regions such as the femur/knee and thorax. Temperature increases that exceed thresholds recommended in safety guidelines and standards are predicted to occur within exposure times up to 15 minutes for all cases except for TrueFISP for abdomen and pelvis imaging at 1.5 T, and GRE at 1.5 T and 3 T, for all imaging regions considered. Although the dependence of RF heating on the relative position of implant and transmit coil has been referenced previously in the literature, this study indicates a need for assessment of GC heating related to the position of the implant within the scanner and the occurrence of maximum change in the gradient fields.

Realistic simulations of more complicated examples, such as clinical protocols that involve a combination of sequences and other types of implant, need to be addressed in future work.

## Supporting information


**FIGURE S1** DUKE model with prosthesis centered relative to the RF coil and gradient coils
**FIGURE S2** Section of the first TR of the turbo spin‐echo (TSE) sequence relevant to a slice signal acquisition. Sixteen RF echoes are clearly visible after the first 90° RF excitation pulse
**FIGURE S3** One acquisition frame of the EPI sequence
**FIGURE S4** Section of the first TR of the gradient‐echo (GRE) sequence relevant to a slice signal acquisition. Spoiling and rewinding gradients are visible along the frequency‐encoding and phase‐encoding directions, respectively
**FIGURE S5** First TR of the true fast imaging sequence with steady precession (TrueFISP) sequence
**FIGURE S6** Radiofrequency influence box (drawn in red) represented for the *xz*, *yz,* and *xy* slices crossing the center of mass (the red dot) of the implant bounding box (drawn in black). The color map represents the value of the normalized ΔS
**FIGURE S7** Comparison between RF heating with and without implant. The results (maps of the temperature increase Δ*T*) refer to 3 T, with the TSE sequence having variable dead time (see Table 2). For each imaging region (thorax, abdomen, pelvis, and femur/knee), the worst‐case position is reported
**FIGURE S8** Results for thorax imaging extending those reported in Figures 3 and 4. Plots show the temperature increase Δ*T* (after 360 seconds) of each voxel belonging to the region of influence versus the minimum distance *d* from the implant surface. Upper figure refers to 1.5 T, whereas the lower figure refers to 3 T. For the TSE sequence, the results are related to a dead‐time variable for each body position (see Table 2). When the same temperature/distance occurs in more than one tissue, earlier points are overwritten
**FIGURE S9** Results for abdomen imaging extending those reported in Figures 3 and 4. Plots show the temperature increase Δ*T* (after 360 seconds) of each voxel belonging to the region of influence versus the minimum distance *d* from the implant surface. Upper figure refers to 1.5 T, whereas the lower figure refers to 3 T. For the TSE sequence, the results are related to a dead‐time variable for each body position (see Table 2). When the same temperature/distance occurs in more than one tissue, earlier points are overwritten
**FIGURE S10** Results for pelvis imaging extending those reported in Figures 3 and 4. Plots show the temperature increase Δ*T* (after 360 seconds) of each voxel belonging to the region of influence versus the minimum distance *d* from the implant surface. Upper figure refers to 1.5 T, whereas the lower figure refers to 3 T. For the TSE sequence, the results are related to a dead‐time variable for each body position (see Table 2). When the same temperature/distance occurs in more than one tissue, earlier points are overwritten
**FIGURE S11** Results for femur/knee imaging extending those reported in Figures 3 and 4. Plots show the temperature increase Δ*T* (after 360 seconds) of each voxel belonging to the region of influence versus the minimum distance *d* from the implant surface. Upper figure refers to 1.5 T, whereas the lower figure refers to 3 T. For the TSE sequence, the results are related to a dead‐time variable for each body position (see Table 2). When the same temperature/distance occurs in more than one tissue, earlier points are overwritten
**TABLE S1** Whole‐body specific absorption rate (W/kg) for the imaging regions (body/coil positions) and sequences. Note: For the TSE sequence, the whole‐body specific absorption rate (SARwb) values are reported for the cases with both a single dead time for all positions (0.30 seconds for 1.5 T and 5.67 seconds for 3 T) and with dead times adjusted to limit the SARwb to 2 W/kg for each body position. The cases scaled to 2 W/kg are denoted in bold. Body positions refer to those defined in Figure 1

## Data Availability

The data sets of results are publicly available on Zenodo (10.5281/zenodo.4049839).
