# Supplementary material for: Heating of hip joint implants in MRI: The combined effect of RF and switched‐gradient fields
Source: Magn Reson Med. 2021 Jan 22;85(6):3447–62. doi: 10.1002/mrm.28666 (PMC7986841; doi:10.1002/mrm.28666)
Supplement: Supplementary file 1 — FIGURE S1 DUKE model with prosthesis centered relative to the RF coil and gradient coils FIGURE S2 Section of the first TR of the turbo spin‐echo (TSE) sequence relevant to a slice signal acquisition. Sixteen RF echoes are clearly visible after the first 90° RF excitation pulse FIGURE S3 One acquisition frame of the EPI sequence FIGURE S4 Section of the first TR of the gradient‐echo (GRE) sequence relevant to a slice signal acquisition. Spoiling and rewinding gradients are visible along the frequency‐encoding and phase‐encoding directions, respectively FIGURE S5 First TR of the true fast imaging sequence with steady precession (TrueFISP) sequence FIGURE S6 Radiofrequency influence box (drawn in red) represented for the xz, yz, and xy slices crossing the center of mass (the red dot) of the implant bounding box (drawn in black). The color map represents the value of the normalized ΔS FIGURE S7 Comparison between RF heating with and without implant. The results (maps of the temperature increase ΔT) refer to 3 T, with the TSE sequence having variable dead time (see Table 2). For each imaging region (thorax, abdomen, pelvis, and femur/knee), the worst‐case position is reported FIGURE S8 Results for thorax imaging extending those reported in Figures 3 and 4. Plots show the temperature increase ΔT (after 360 seconds) of each voxel belonging to the region of influence versus the minimum distance d from the implant surface. Upper figure refers to 1.5 T, whereas the lower figure refers to 3 T. For the TSE sequence, the results are related to a dead‐time variable for each body position (see Table 2). When the same temperature/distance occurs in more than one tissue, earlier points are overwritten FIGURE S9 Results for abdomen imaging extending those reported in Figures 3 and 4. Plots show the temperature increase ΔT (after 360 seconds) of each voxel belonging to the region of influence versus the minimum distance d from the implant surface. Upper figure refers to 1.5 T, wherea [file MRM-85-3447-s001.docx]

**Supporting Information**

**Supporting Information Section S1 - RF Birdcage, Gradient coils, and Prosthesis**


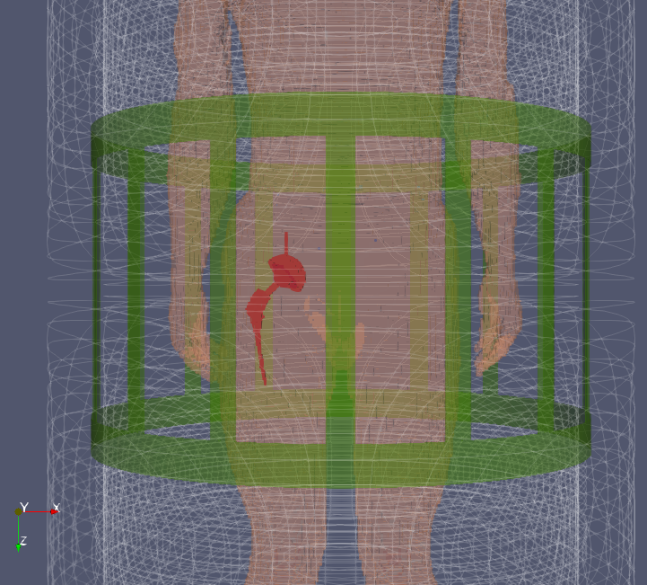


Supporting Information Figure S1 – DUKE model with prosthesis centred relative to the RF coil and GCs.

**Supporting Information Section S2 - Time waveforms of the selected MRI sequences**

The time waveforms of the four selected MRI sequences, whose parameters are collected in Table 2, are shown in Supporting Information Figures S2 to S5. The amplitude of the RF pulses is shown in the upper panels, whilst the bottom panels show the gradient along the frequency encoding (G_FE_), phase encoding (G_PE_) and slice selection (G_SS_) directions. Crusher gradients have been designed along the frequency and slice selection directions to reduce the unwanted transverse magnetization caused by the imperfect refocusing slice profile.


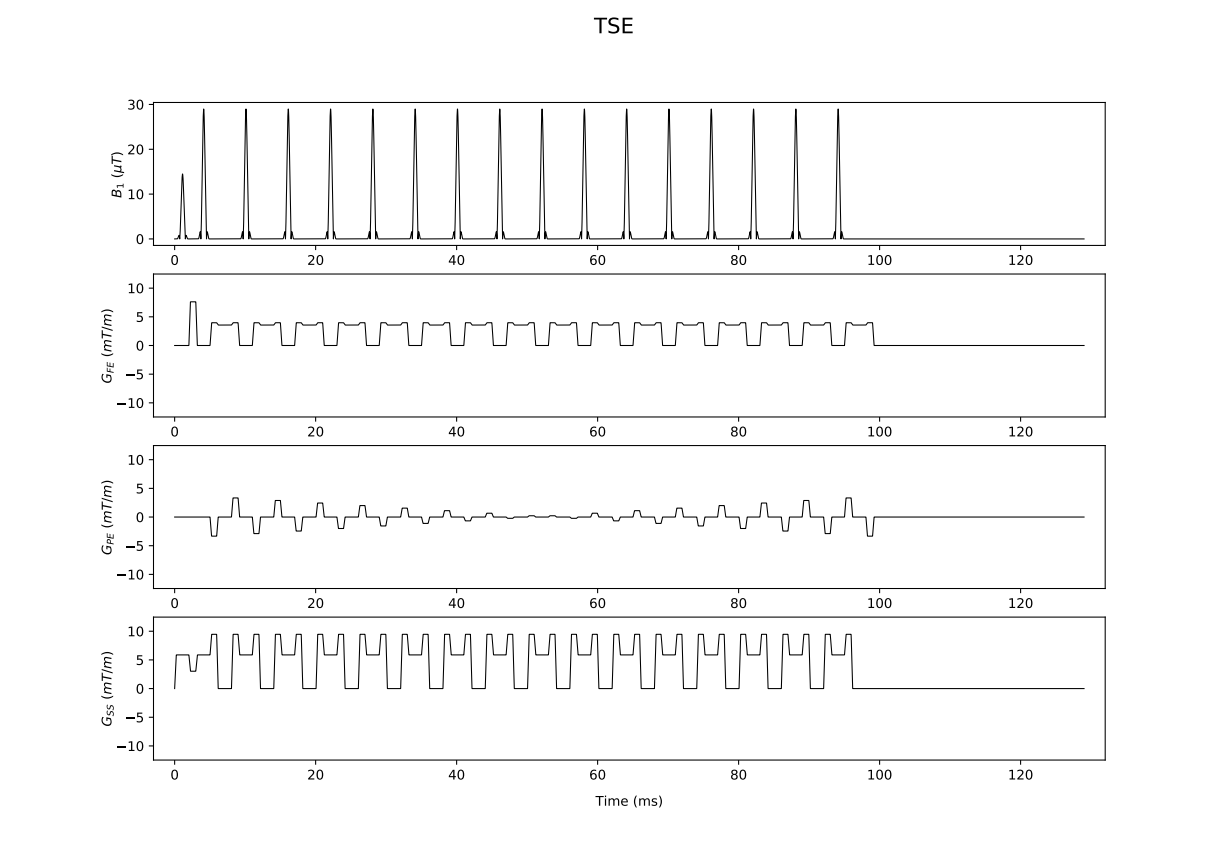


Supporting Information Figure S2 – Section of the first Repetition Time (TR) of the TSE sequence relevant to a slice signal acquisition. 16 RF echoes are clearly visible after the first 90° RF excitation pulse.


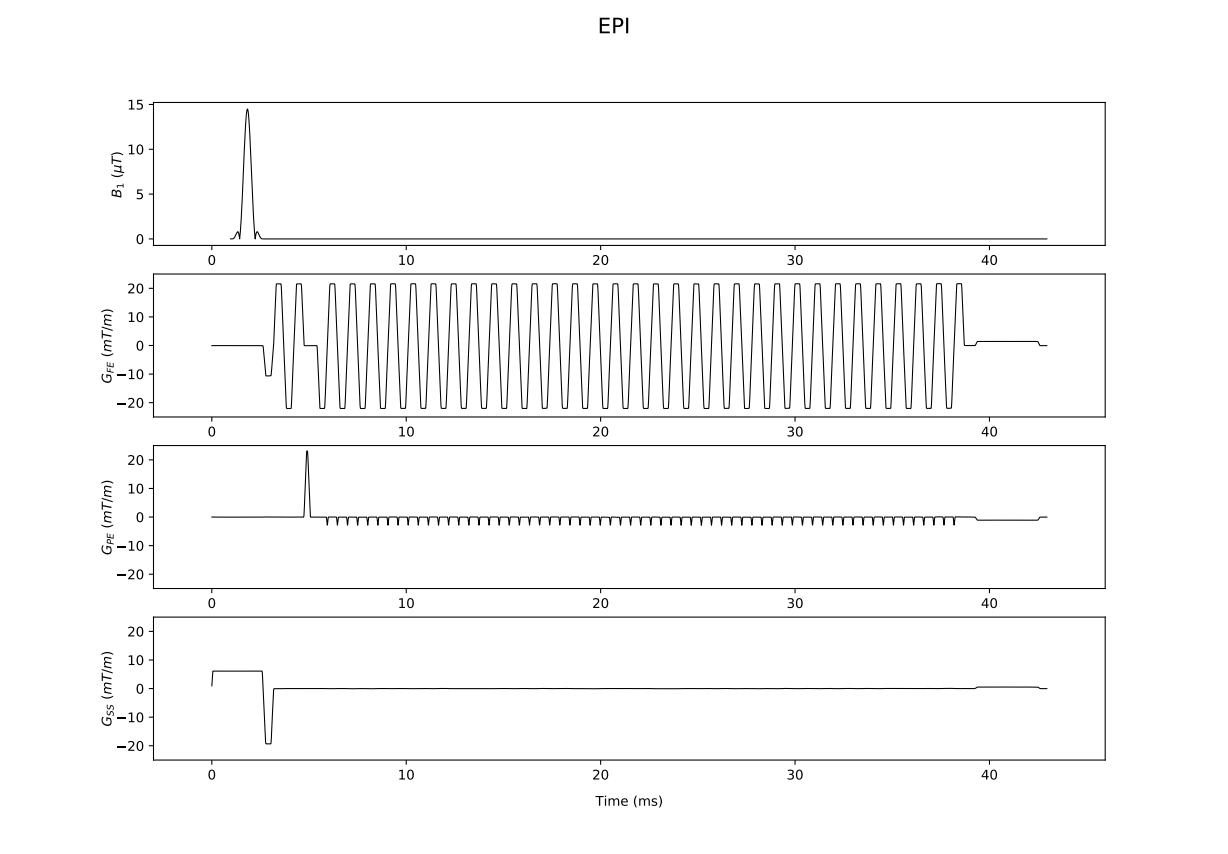


Supporting Information Figure S3 – One acquisition frame of the EPI sequence.


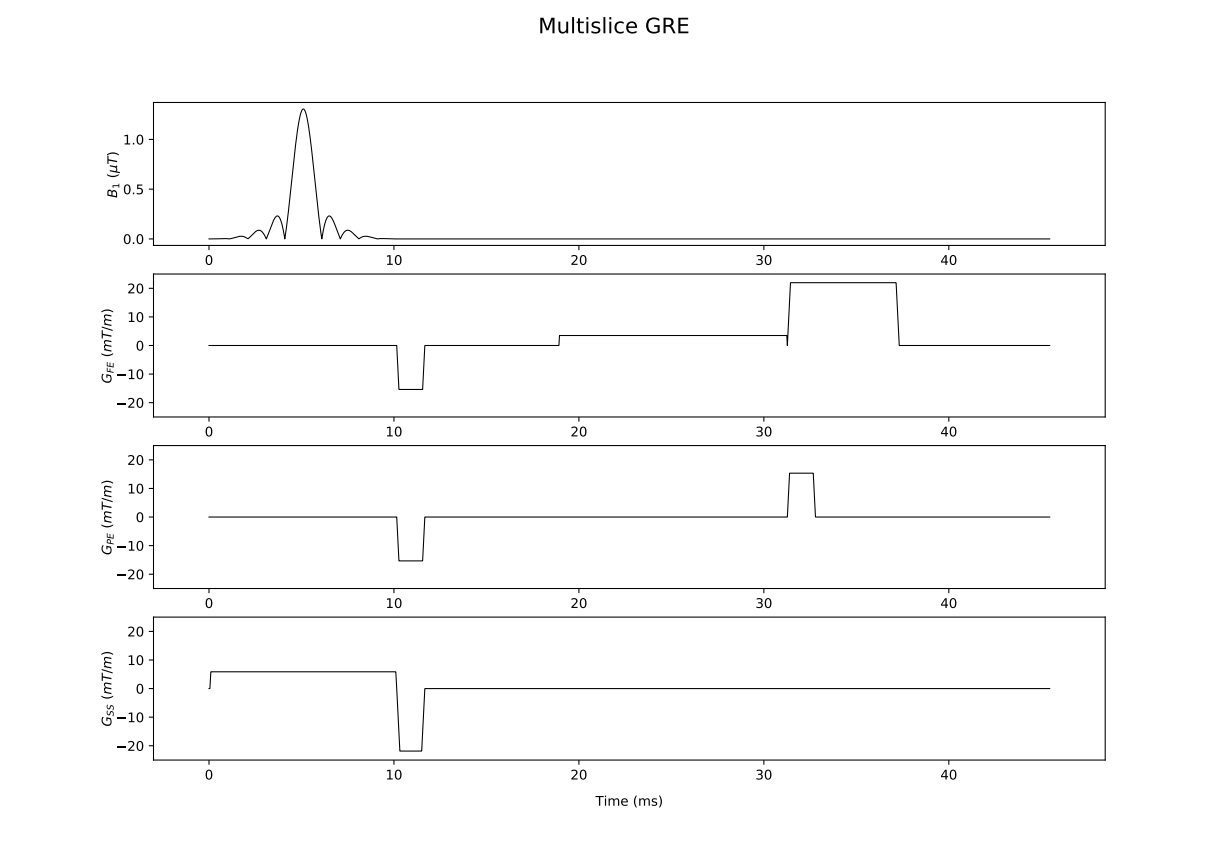


Supporting Information Figure S4 – Section of the first Repetition Time (TR) of the GRE sequence relevant to a slice signal acquisition. Spoiling and rewinding gradients are visible along the frequency and phase encoding direction respectively.


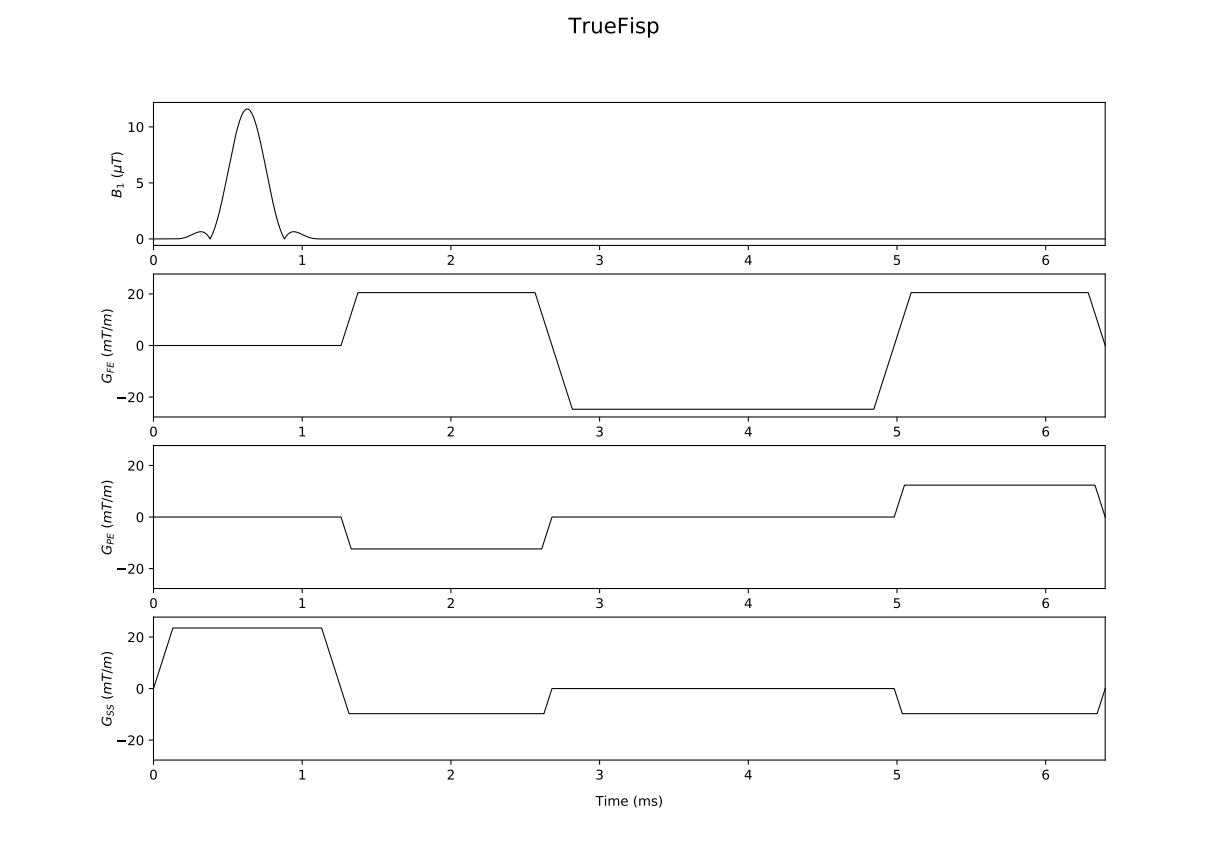


Supporting Information Figure S5 – First Repetition Time (TR) of the TrueFISP sequence.

**Supporting Information Section S3 - Whole-body SAR (SARwb)**

The SARwb computed for each position and for the four sequences considered at 1.5 T and 3 T is shown in Supporting Information Table S1.

Supporting Information Table S1 –SARwb (W/kg) for the imaging regions (body/coil positions) and sequences. For the TSE sequence, the SARwb values are reported for the cases with both a single dead time for all positions (0.30 s for 1.5 T and 5.67 s for 3 T) and with dead times adjusted to limit the SARwb to 2 W/kg for each body position. The cases scaled to 2 W/kg are denoted in **bold**. Body positions refer to those defined in Figure 1.

| MRI Region | **1.5 T** | | | | | | | | | | **3 T** | | | | | | | | | |
| --- | --- | --- | --- | --- | --- | --- | --- | --- | --- | --- | --- | --- | --- | --- | --- | --- | --- | --- | --- | --- |
|  | **FISP** | | **EPI** | | **TSE with unique dead time** | | **TSE with variable dead time** | | **GRE** | | **FISP** | | **EPI** | | **TSE with unique dead time** | | **TSE with variable dead time** | | **GRE** | |
|  | Body Pos. | wbSAR (W/kg) | Body Pos. | wbSAR (W/kg) | Body Pos. | wbSAR (W/kg) | Body Pos. | wbSAR (W/kg) | Body Pos. | wbSAR (W/kg) | Body Pos. | wbSAR (W/kg) | Body Pos. | wbSAR (W/kg) | Body Pos. | wbSAR (W/kg) | Body Pos. | wbSAR (W/kg) | Body Pos. | wbSAR (W/kg) |
| Thorax | 1 | 0.29 | 1 | 0.11 | 1 | **2.00** | 1 | **2.00** | 1 | 0.0024 | 1 | 0.93 | 1 | 0.35 | 1 | **2.00** | 1 | **2.00** | 1 | 0.026 |
| Abdomen | 5 | 0.21 | 5 | 0.077 | 5 | 1.48 | 5 | 1.70 | 3 | 0.0019 | 4 | 0.78 | 3 | 0.28 | 4 | 1.69 | 4 | **2.00** | 3 | 0.021 |
| Pelvis | 6 | 0.20 | 8 | 0.061 | 8 | 1.14 | 8 | 1.31 | 8 | 0.0013 | 8 | 0.64 | 8 | 0.24 | 8 | 1.38 | 8 | **2.00** | 8 | 0.018 |
| Femur/  knee | 11 | 0.10 | 10 | 0.045 | 9 | 1.00 | 9 | 1.14 | 11 | 0.0008 | 9 | 0.54 | 10 | 0.17 | 9 | 1.16 | 9 | **2.00** | 10 | 0.013 |

**Supporting Information Section S4 - Rationale behind the choice of the region of influence around the implant**

The analysis of the results was deepened in a parallelepiped box (region of influence) defined as a portion of the body volume in which the presence of the metallic implant sensibly modifies the electromagnetic and thermal fields. General considerations, deduced from preliminary computations performed on a phantom, clearly showed that the portion of volume affected by the presence of the prosthesis is maximised for the RF fields at 123.2 MHz (3 T) during the pelvis imaging. Such conditions were assumed for the identification of the region of influence, performed on the anatomical body model. Each voxel *i* belonging to the body was associated to the quantity $\Delta S\left( i \right)=\left| {SAR}_{imp}\left( i \right)-{SAR}_{no}\left( i \right) \right|$, being *SAR*_imp_ and *SAR*_no_ the SAR values in the voxel *i* with and without the implant, respectively. The voxels occupying the volume of the implant were not accounted for. The differences ΔS(*i*), normalized with respect to its maximum value, were adopted as variation index. The region of influence was defined as a parallelepiped which contains the bounding box of the implant and includes all the voxels whose variation index exceeds a stated threshold.

Assuming a 7 % threshold, the region of influence is a box with dimensions: 21.7 cm × 18.8 cm × 28.2 cm. In Supporting Information Figure S6, the region of influence is represented by the red rectangle. The figure shows the *xz*, *yz* and *xy* slices which cross the centre of mass of the implant bounding box. The value of the variation index is reported according to the colour bar. The volume of the region of influence is about 16 % of the whole volume of the body, and the L2 norm of Δ*S* extended to the region of influence is about 90 % of the L2 norm extended to the entire body.


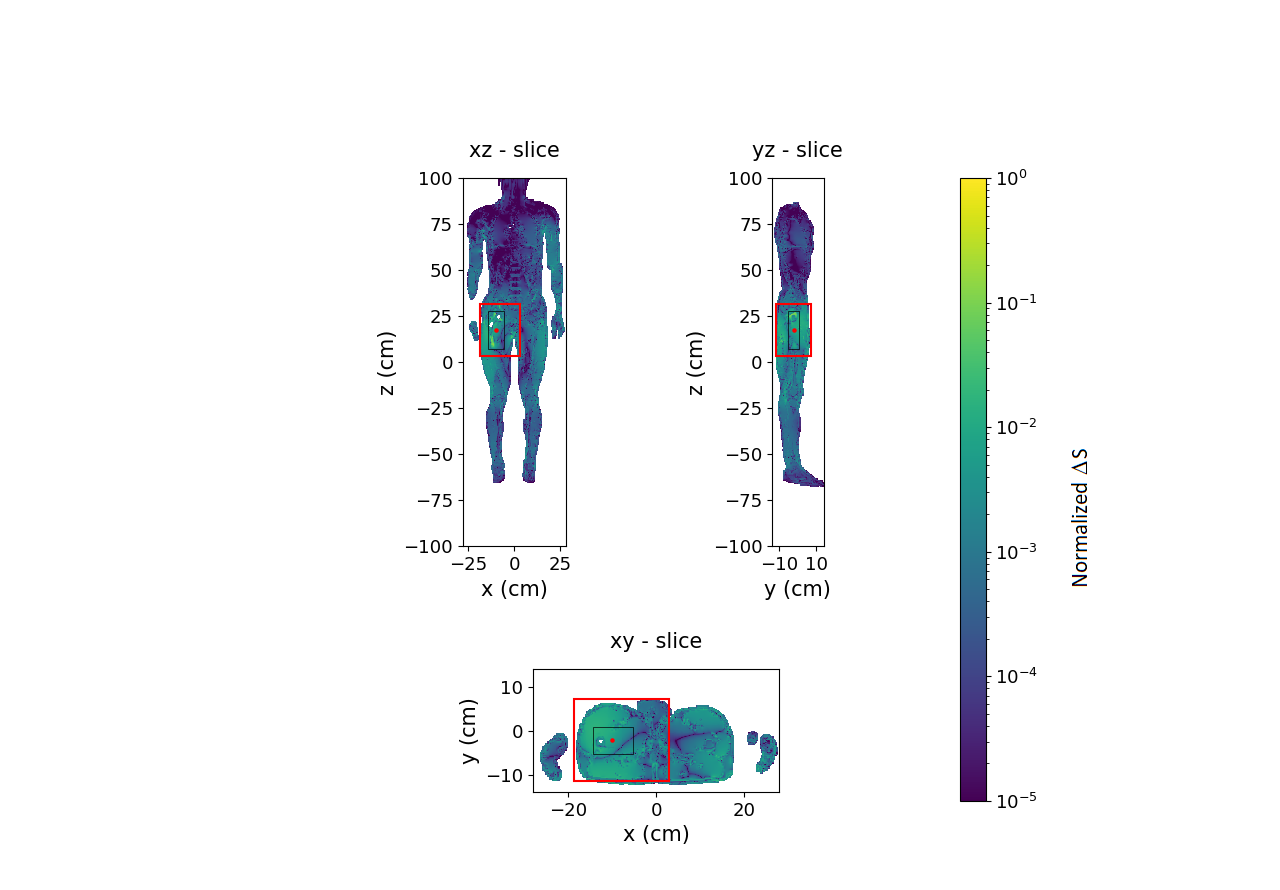


Supporting Information Figure S6 – RF influence box (drawn in red) represented for the *xz*, *yz* and *xy* slices crossing the centre of mass (the red dot) of the implant bounding box (drawn in black). The colour map represents the value of the normalized ΔS.

**Supporting Information Section S5 - Rationale behind the choice of the thresholds for temperature increase**

Both the IEC Standard 60601-2-33 [8] and the recent ICNIRP Guidelines [42] set safety thresholds expressed in terms of temperature. In particular, the IEC recommends that, in normal operating mode, both the core and local temperatures keep below 39 °C, whereas ICNIRP treats local temperature of 41 °C (or greater) as potentially harmful. Since in this paper the analysis was developed in terms of temperature increase, the choice of the corresponding safety thresholds required the preliminary evaluation of the distribution of the physiological temperature at steady state in the body in the absence of any electromagnetic exposure. Such an analysis was performed on the ‘Duke’ model without implant, using a thermal solver based on the stationary version of Pennes’ bioheat equation, with temperature-independent parameters. Several simulations were carried out, changing the values of the blood temperature (between 37 °C and 37.5 °C), the external ambient temperature (between 20 °C and 25 °C), and the coefficient of heat exchange between the skin and the external environment. In all simulations, the minimum temperature occurred in the skin, particularly in the ears, and the maximum temperature was always localized in the cardiac region; its value was ~0.6 K higher than the blood temperature, independently of the other two parameters. The resulting average temperature, evaluated over all tissues, was slightly lower than the blood temperature. The temperature of deep tissues was almost uniform and close to the blood temperature, but exhibited slightly higher local peaks. Within the region of influence around the implant, the average and maximum temperatures were 0.2 K lower and 0.3 K larger than the blood temperature, respectively. Hence, if the blood temperature was set to 37.5 °C, the maximum temperature in such regions amounted to 37.8 °C. Based on this result and adopting a conservative approach, a maximum local physiological temperature equal to 38 °C was taken as a starting point. With this assumption, the reference thresholds adopted throughout the paper, equal to 1 K and 3 K, therefore correspond to the temperature increase needed to reach the maximum temperatures allowed by IEC and ICNIRP, respectively.

The bioheat equation provides a conveniently easy way to model the thermal balance in the body at the cost of some physical inaccuracies. In particular, the total metabolic power in the simulated human model amounted to ~126 W. At steady state, this power should be perfectly balanced by the thermal flux that crosses the skin towards the external environment and the blood should simply “move” the heat, whilst conserving it. However, in most simulations the global thermal flux across the skin was predicted to be larger than the metabolic heat. This inconsistency implicitly arises from the simplifying assumption of temperature-independent parameters in the model and is the cost of using a relatively simple mathematical description.

**Supporting Information Section S6 - Additional results related to the implant and tissue heating**


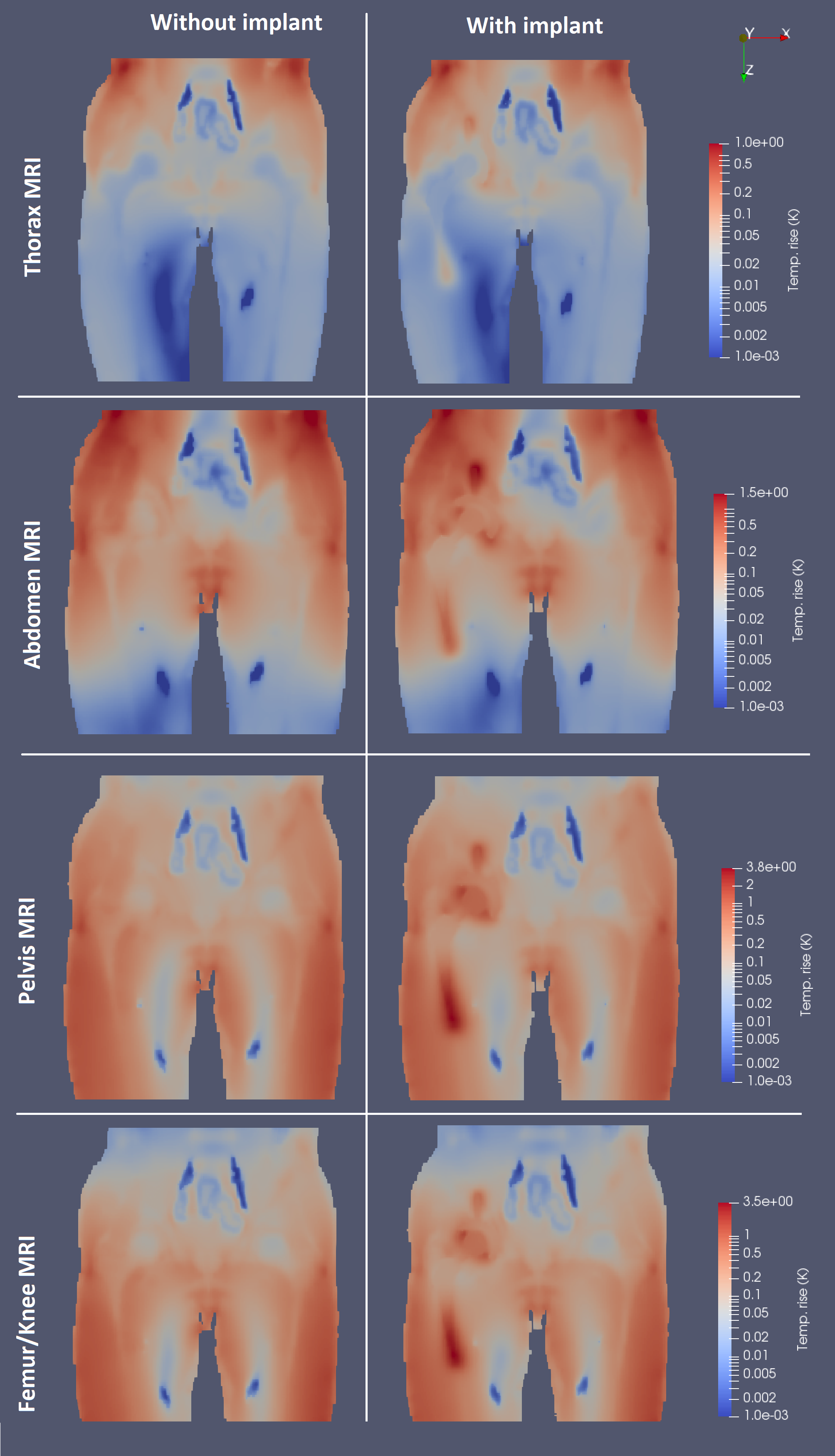


Supporting Information Figure S7 – Comparison between RF heating with and without implant. The results (maps of the temperature increase Δ*T*) refer to 3 T with TSE sequence having variable dead time (see Table 2). For each imaging region (thorax, abdomen, pelvis, femur/knee) the worst-case position is here reported.


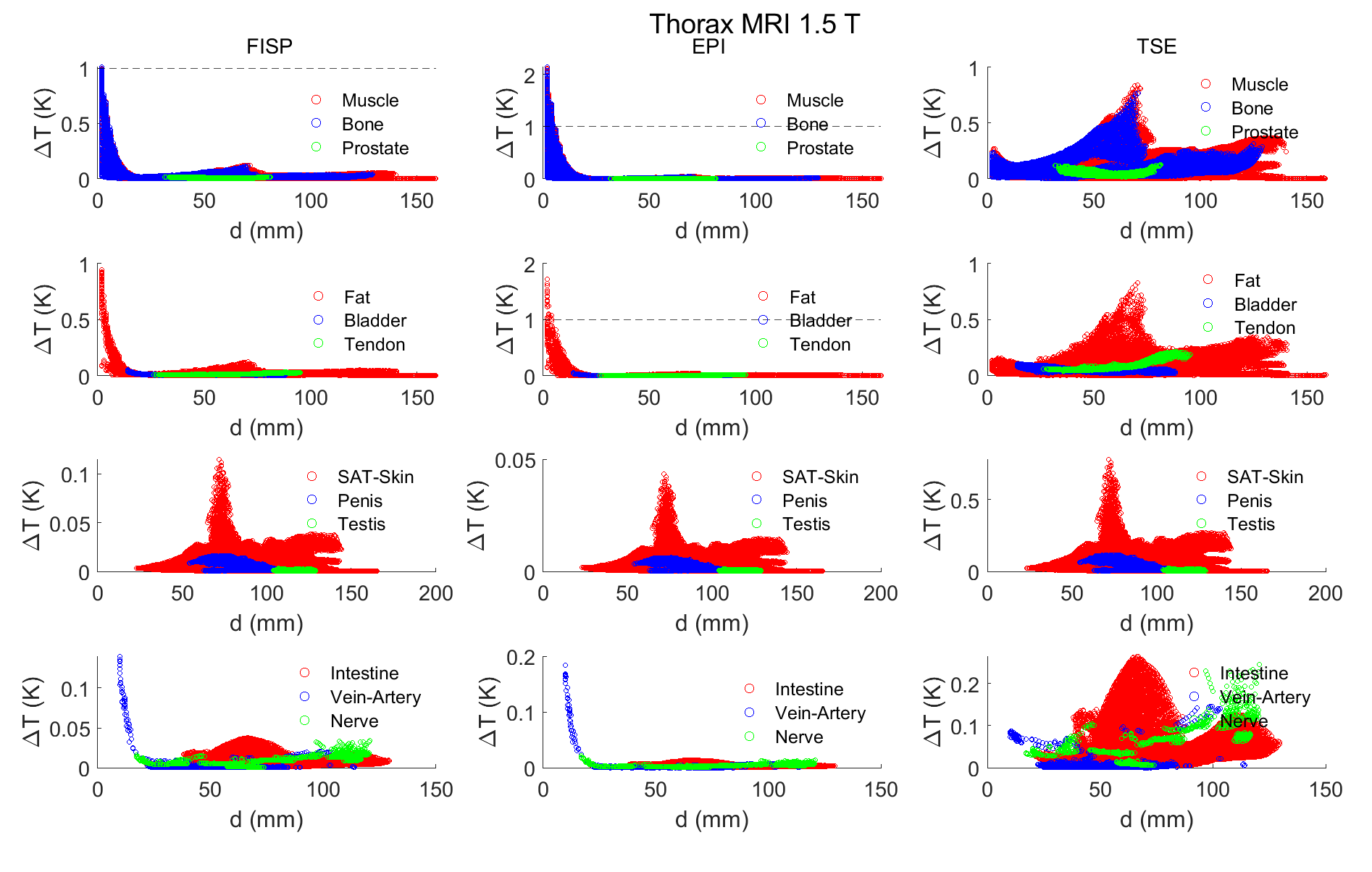


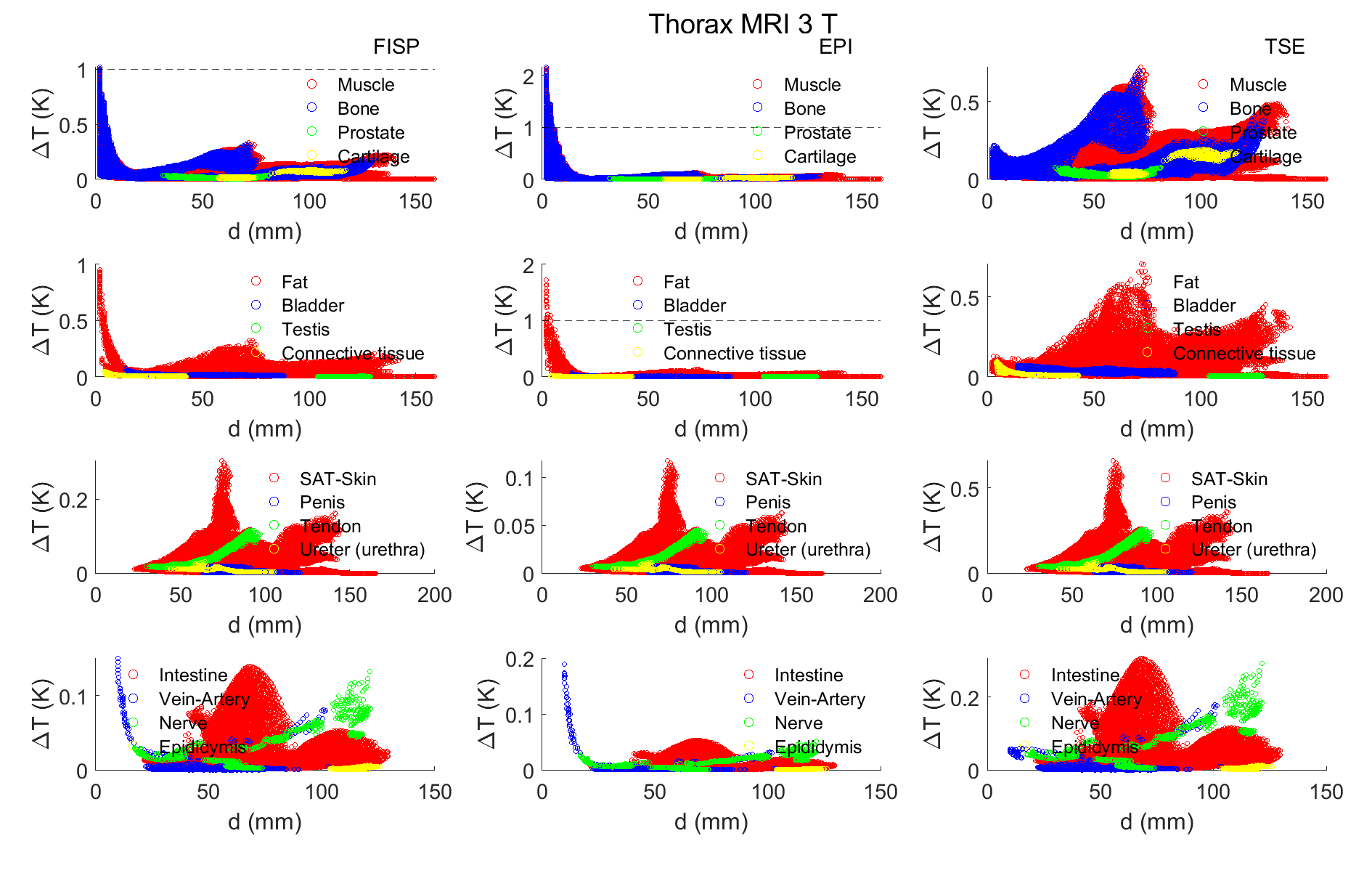


Supporting Information Figure S8 – Results for thorax imaging extending those reported in Figures 3 and 4. Plots of the temperature increase Δ*T* (after 360 s) of each voxel belonging to the region of influence versus the minimum distance d from the implant surface. Upper figure refers to 1.5 T, while the lower figure refers to 3 T. For TSE sequence, the results are related to a dead time variable for each body position (see Table 2). When the same temperature/distance occurs in more than one tissue, earlier points are overwritten.


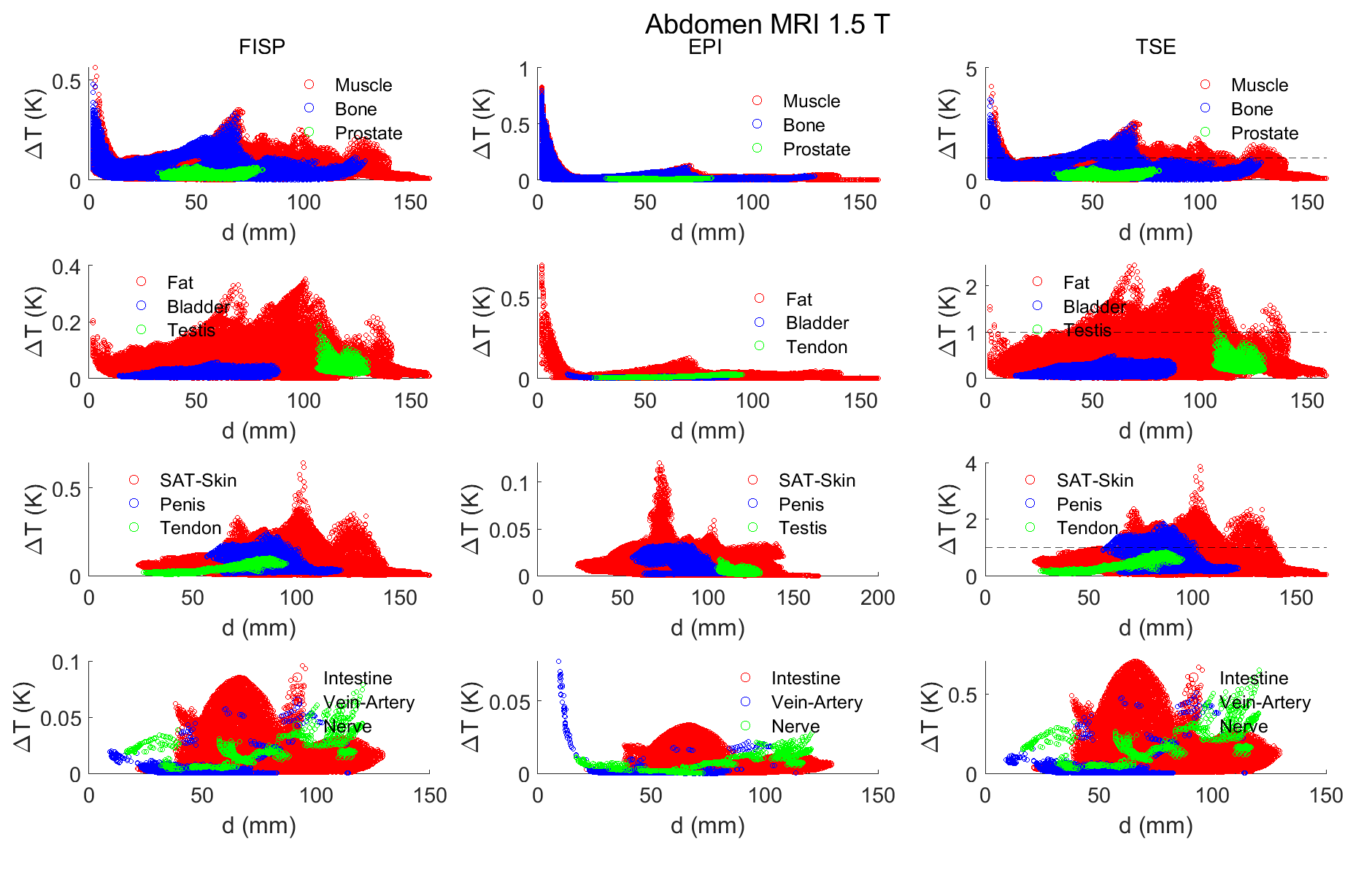


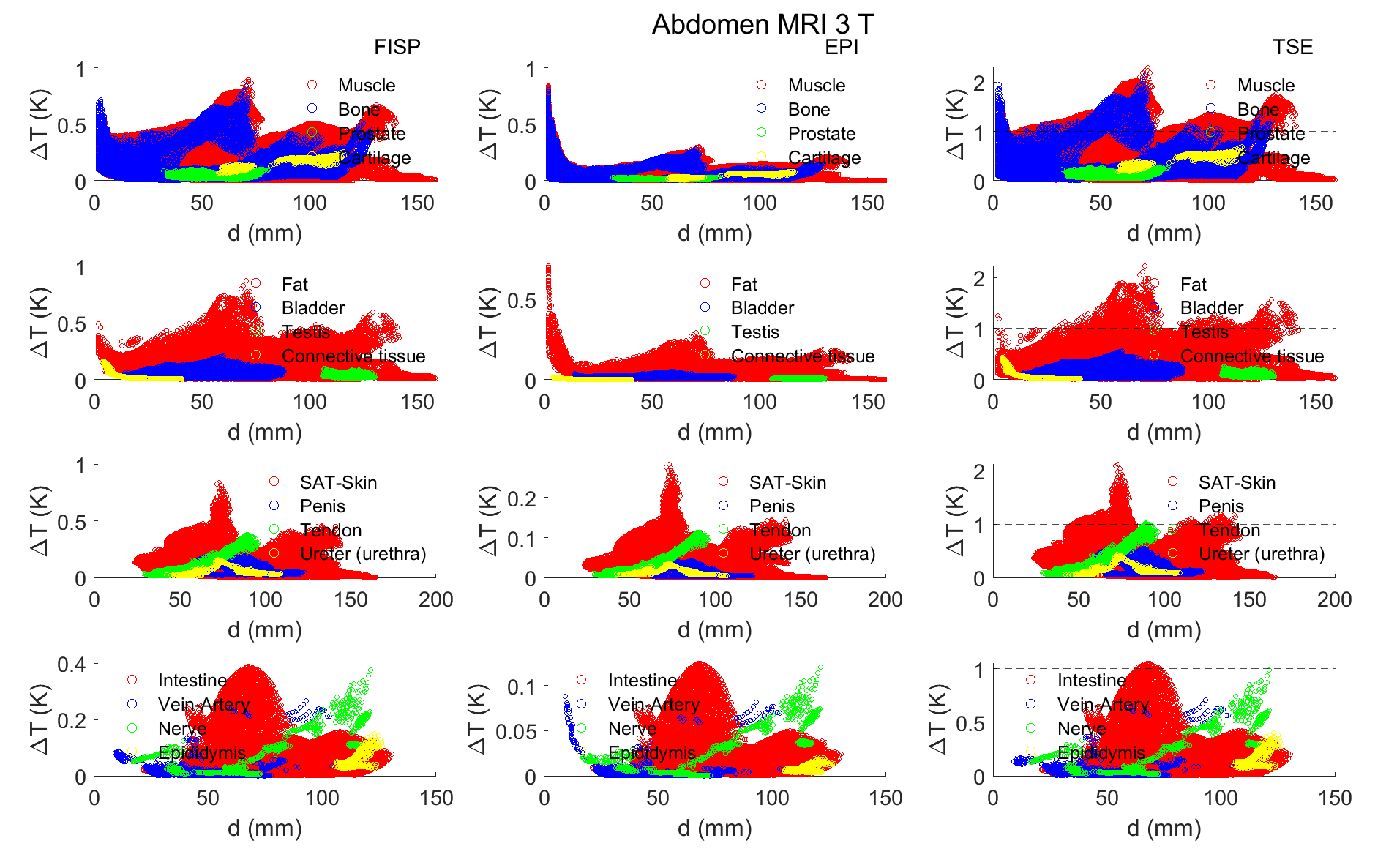


Supporting Information Figure S9 – Results for abdomen imaging extending those reported in Figures 3 and 4. Plots of the temperature increase Δ*T* (after 360 s) of each voxel belonging to the region of influence versus the minimum distance d from the implant surface. Upper figure refers to 1.5 T, while the lower figure refers to 3 T. For TSE sequence, the results are related to a dead time variable for each body position (see Table 2 When the same temperature/distance occurs in more than one tissue, earlier points are overwritten.


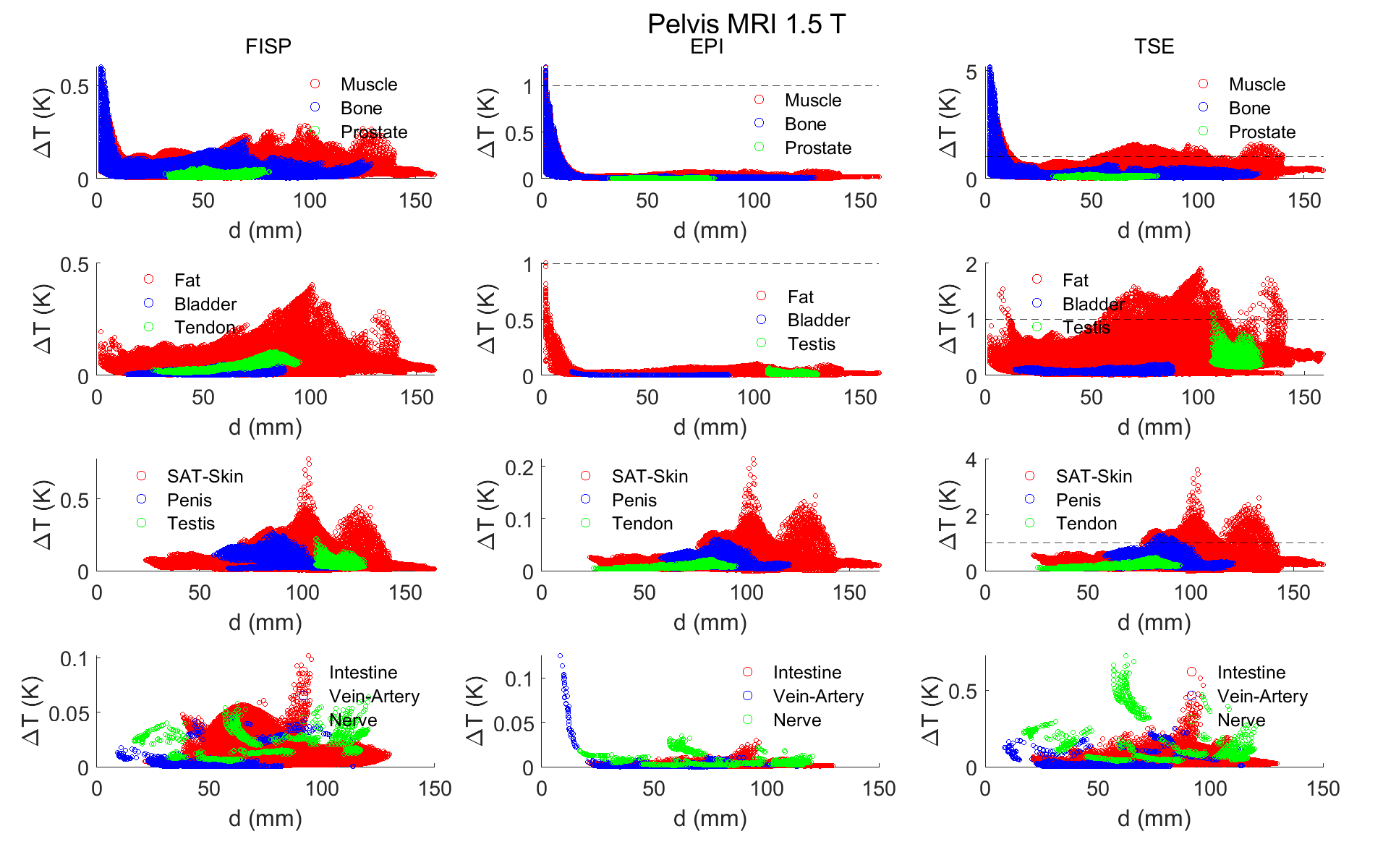


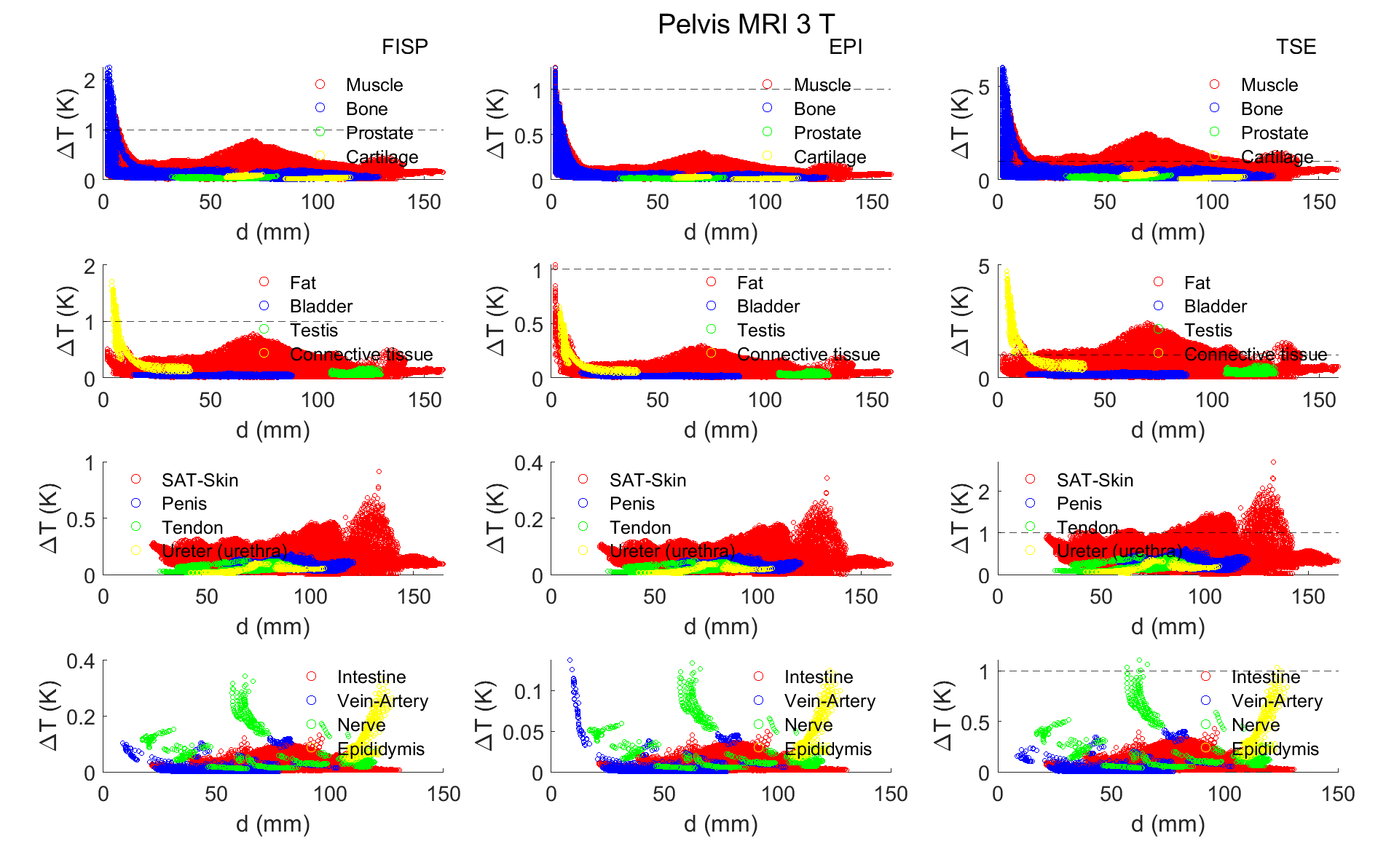


Supporting Information Figure S10 – Results for pelvis imaging extending those reported in Figures 3 and 4. Plots of the temperature increase Δ*T* (after 360 s) of each voxel belonging to the region of influence versus the minimum distance d from the implant surface. Upper figure refers to 1.5 T, while the lower figure refers to 3 T. For TSE sequence, the results are related to a dead time variable for each body position (see Table 2). When the same temperature/distance occurs in more than one tissue, earlier points are overwritten.


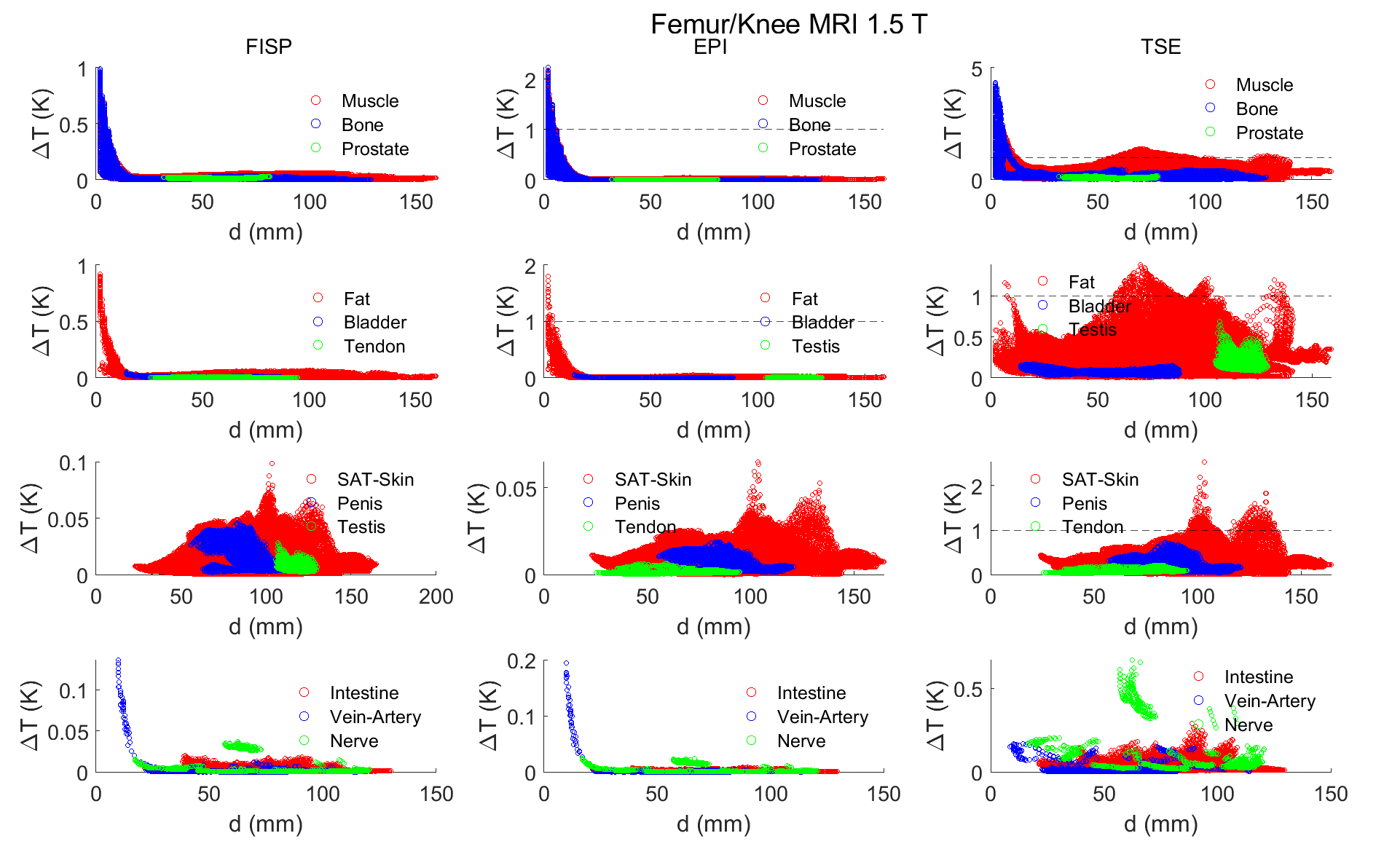


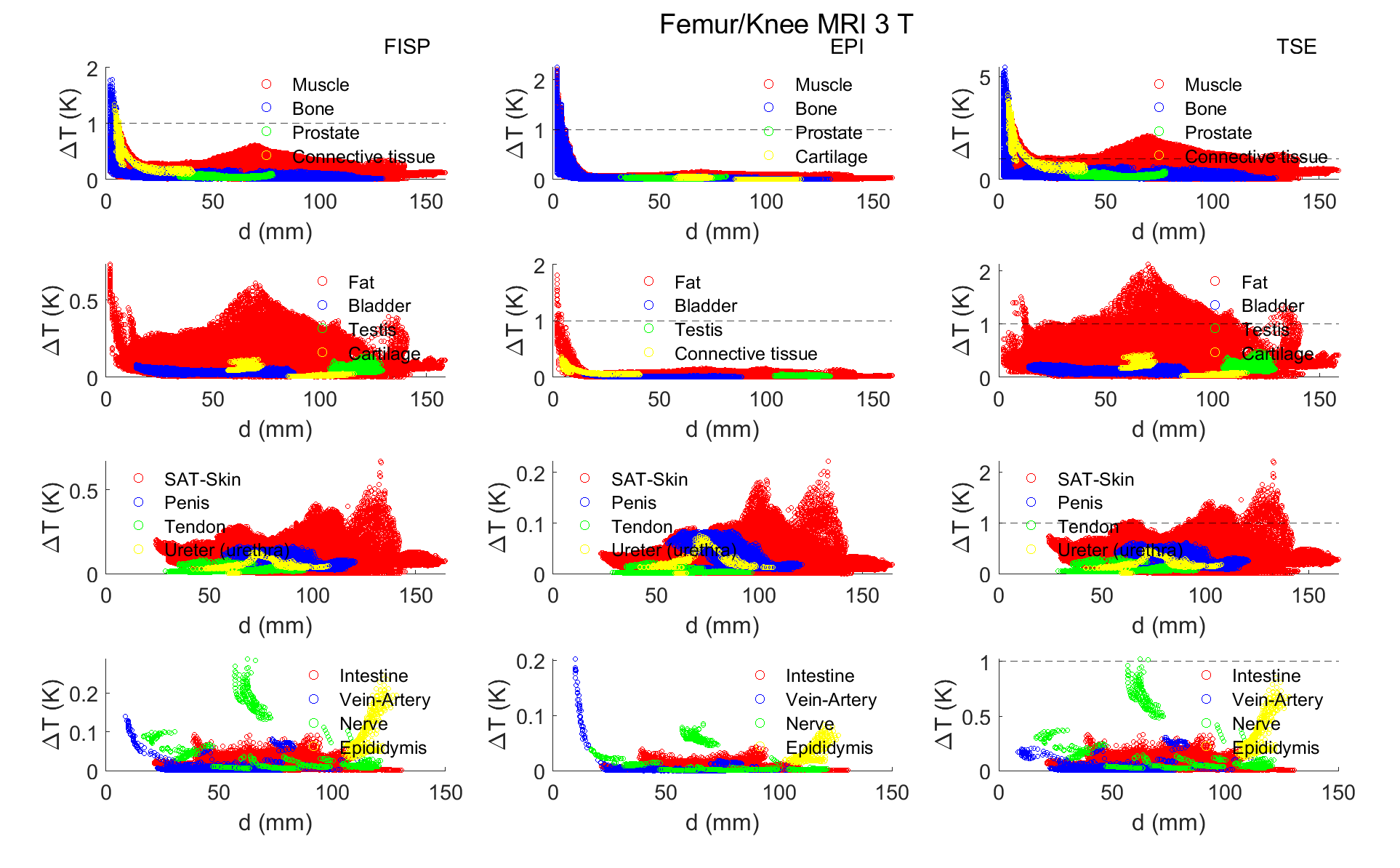


Supporting Information Figure S11 – Results for femur/knee imaging extending those reported in Figures 3 and 4. Plots of the temperature increase Δ*T* (after 360 s) of each voxel belonging to the region of influence versus the minimum distance d from the implant surface. Upper figure refers to 1.5 T, while the lower figure refers to 3 T. For TSE sequence, the results are related to a dead time variable for each body position (see Table 2). When the same temperature/distance occurs in more than one tissue, earlier points are overwritten.
